# *Ab initio* word recognition in infant- and adult-directed continuous speech

**DOI:** 10.1017/s0142716425100350

**Published:** 2025-12-29

**Authors:** Katie Von Holzen, Rochelle S. Newman

**Affiliations:** 1 English and American Studies, Technische Universität Braunschweig, Braunschweig, Germany; 2 Department of Hearing and Speech Sciences, University of Maryland, College Park, MD, USA

**Keywords:** *Ab initio* learners, first exposure, infant-directed speech, word recognition

## Abstract

Continuous speech presents a challenge to the *ab initio* learner, as the language-specific segmentation strategies they use in their first language are not always reliable cues in other languages (Cutler 2001 *International Journal of Research and Practice in Interpreting*, 5(1), 1–23). Yet, they are able to use more general acoustic, prosodic, and statistical cues to word boundaries, as well as lexical similarity to their first language (e.g., Shoemaker & Rast 2013. *Second Language Research*, 29(2), 165–183) to recognize words at first exposure to a new language. In the current study, we investigated whether adult *ab initio* learners’ ability to recognize words after brief exposure to continuous speech in a new language is improved when that speech is produced using an infant-directed register, a style of speech found to facilitate segmentation in infancy (Thiessen et al. 2005. *Infancy* 7(1), 53–71). In a series of experiments, we demonstrate that although English *ab initio* learners of German benefited from infant-directed speech, their performance was generally lower than in previous studies unless task demands were reduced. These learners also benefited from word length and from frequency of occurrence, as has been shown previously, but these did not interact with register. As in infancy, learner-directed speech registers appear to facilitate initial processing and recognition in adult *ab initio* learners.

## Introduction

Many modern foreign language learning classrooms use immersive techniques to teach foreign languages, which include speaking exclusively in the to-be-learned language. In these immersive environments, it is likely that the teacher will not produce words in isolated instances with pauses in between, but instead use continuous utterances. Continuous speech presents a particular challenge to these beginning language learners, as the continuous input has to be segmented into individual words before meaning can be attached to the segmented sound strings. This has direct consequences for processing, as *ab initio* learners’ recognition of words in continuous speech is delayed and their electrophysiological responses reduced in comparison to native speakers (Snijders et al., 2007). In the current study, we examine the conditions under which initial word recognition can be facilitated in adult *ab initio* learners, focusing on the prosodic modifications present in infant-directed speech.

Listeners’ approach to recognizing words in continuous speech is highly language-specific and is influenced by the language’s prosodic, phonological, and lexical structure (Cutler, 2001; Mattys & Bortfeld, 2016). Given the same input, native listeners of a stress-based language will segment it into words that match the input’s stress pattern, while native listeners of a syllable-based language will segment it into words that match the input’s syllable pattern (Cutler et al., 1986; Mehler et al., 1981). The first language continues to exert an influence on strategies in subsequently acquired languages; even highly proficient L2 users continue to apply cues specific to their L1, even when these L1 cues are inappropriate for the context (Hanulíková et al., 2011; Weber & Cutler, 2006; see also Finn & Hudson Kam, 2008).

The *ab initio* learner ideally should therefore not rely on the same strategies that they use to recognize words in the speech of their native language. Although *ab initio* learners will continue to apply these incorrect L1 strategies, they have also been found to rely on more general, acoustic or prosodic cues to word boundaries. For example, words that occur near utterance boundaries are more readily recognized (e.g., Shoemaker & Rast, 2013), potentially due to a word boundary being marked by silence. The frequency of occurrence of a particular word structure in the native language may also facilitate its segmentation and recognition in a new language. Dutch *ab initio* learners more readily recognize bisyllabic compared with monosyllabic words from Mandarin Chinese (Gullberg et al., 2012), possibly because bisyllabic words are more frequent than monosyllabic words in Dutch (Vroomen et al., 1996) or because bisyllabic words have a trochaic stress pattern in Mandarin (Lai et al., 2010) and initial stress is a cue for word boundaries in Dutch (e.g., Vroomen et al., 1996).

*Ab initio* listeners begin to form lexical knowledge about a new language after only a brief exposure. Gullberg and colleagues (Gullberg et al., 2012) found that words that occurred more frequently in the input were more accurately recognized by novice listeners than infrequently occurring words. Frequency of occurrence in the input is thought to modulate the formation of a memory trace (Robinson, 2005). But beyond the frequency with which a form is encountered in the input, listeners are also sensitive to the statistical co-occurrence of syllables present in the input. They have been found to use the transitional probability between syllables, that is, the probability that syllable X will be followed by syllable Y or vice versa, to determine the boundaries between words after only a few minutes of exposure (Saffran et al., 1997). The evidence for the use of transitional probabilities has been demonstrated across the lifespan; adults, children, and infants as young as 5.5 months have been found to track the transitional probability between syllables in an artificial language (Johnson & Tyler, 2010; Saffran et al., 1996). Although many studies investigating the role of statistical learning in initial speech segmentation have used artificial languages, both adults (Alexander et al., 2023; Kittleson et al., 2010) and infants (Pelucchi et al., 2009) have been found to track transitional probabilities in natural speech in a new language as well.

Thus, although initial word recognition provides a challenge to adult *ab initio* learners, they are able to exploit cues available in the input and form word-level representations after brief exposure. In the current study, we examine whether a type of input readily available for infant language learners similarly facilitates the subsequent formation of lexical knowledge in adult *ab initio* learners. Infant- or child-directed speech (IDS; CDS) describes the speech register used when adults are addressing infants and children. It differs from adult-directed speech (ADS) in acoustic characteristics, such as slower speech rate, higher and more varied pitch, and vowel hyperarticulation, as well as structural characteristics, such as simplified phrases and increased repetition (e.g. Fernald, 1992; see Soderstrom, 2007 for a review). IDS has been proposed to support infants’ language acquisition by attracting infants’ attention to the speech signal (Rose et al., 2003) or through the modifications themselves, as infants’ word recognition is facilitated when words are presented in simple, repetitive word sequences, like those found in IDS (Fernald & Cummings, 2003). The simplified phrasal structure and exaggerated prosody could also provide redundant grammatical cues, which would also support learning (e.g. Fisher & Tokura, 1996; Morgan et al., 1987). Infants show enhanced word learning when the input is presented in IDS compared with ADS (Graf Estes & Hurley, 2013; Ma et al., 2011; Song et al., 2010; Zhou et al., 2023), suggesting that the use of IDS has important implications for language learning.

IDS may also be beneficial for speech segmentation and word recognition. IDS often presents longer pauses at phrasal boundaries than ADS (Fernald & Mazzie, 1991) and phrases produced in IDS have on average fewer words than those produced in ADS, which can result in a slower speech rate, a greater percentage of words in the utterances already having one word boundary marked, and more opportunities for phrase-final lengthening (Martin et al., 2016). Although not universally present, such features could aid segmentation (e.g., Gout et al., 2004; White et al., 2020). Pitch peaks are also higher in IDS in comparison to ADS (Fernald et al., 1989). Pitch peaks are a reliable indicator of stress (Jessen et al., 1995; Sluijter & Van Heuven, 1996), which in turn serves as a word boundary cue in stress-based languages (Cutler, 1994) and both English- and German-learning infants successfully use the dominant stress pattern, the trochaic unit, to segment and recognize words (Höhle et al., 2009; Jusczyk et al., 1999). However direct comparisons of the influence of IDS and ADS speech on segmentation ability in infants have yielded mixed results. Singh and colleagues (Singh et al., 2009) found that previous exposure to words produced in English IDS, but not ADS, led to successful word recognition 24 hours later. In contrast, infants learning a language other than English, German, are equally successful at recognizing words presented in IDS and ADS, although this may be due to the language-specific characteristics of German IDS (Mani & Paetzold, 2016; Schreiner et al., 2016).

Infants can also more easily exploit the statistical cues present in speech when it is produced using an IDS as opposed to an ADS register, supporting word segmentation. Thiessen et al. (2005) familiarized English-learning 7-month-olds to an artificial language. Half of the infants heard the language produced with an IDS register, while the other half heard it produced with an ADS register, with recordings modified to have the same overall amplitude and length as well as the occurrence of within-word pitch accents. This ensured that the differences between the IDS and ADS stimuli were due to their pitch contour and fundamental frequency (F0). Infants listened longer to words compared to part words only when they were familiarized with IDS, suggesting that the exaggerated pitch and intonation in this register support speech segmentation.

IDS may be beneficial for adult *ab initio* learners. In a series of experiments, Golinkoff and Alioto (1995) familiarized English-speaking adults with pictures paired with sentences in Chinese labeling the picture (Zhe shi yi ge ping—This is a bottle). Performance at word learning was higher than chance only when participants were familiarized with IDS, but not ADS speech. The word-learning studies of Golinkoff and Alioto (1995) and a similar study of Ma et al. (2020) require the learner to first segment the words from the familiarization sentences and then map the word onto the referential object they were viewing at the time. Although these results suggest that IDS is helpful, it is not clear whether this advantage occurs during segmentation, label-referent association, or both. Indeed, Ma and colleagues found that familiarizing participants with the targets produced in IDS in isolation led to better subsequent word recognition than targets produced in ADS, suggesting that facilitation is at least happening during label-referent association. It therefore remains unclear to what extent IDS aids adult learners in word recognition when the component of label-referent association is removed.

In the current study, we examine whether the exaggerated pitch changes found in infant-directed speech influence adult *ab initio* learners’ ability to recognize words they have been exposed to in a new, natural language. Using a modification of the paradigm used by Gullberg et al. (2012), we compared English-speaking adults’ ability to recognize word forms that were previously presented in continuous speech produced using an IDS or ADS register (target items). Although IDS is typically produced more slowly than ADS, we controlled for this rate difference, such that speaking rate/duration differences were unable to impact recognition. To be more compatible with previous literature, we analyze participants’ accuracy in the form of word acceptance, or their likelihood to indicate that they previously heard a test item during exposure (Gullberg et al., 2012; Kittleson et al., 2010). We also analyzed participants’ *d*’ scores to capture their sensitivity to the presence of target items (Hautus et al., 2021). We predicted that if IDS facilitates word recognition in *ab initio* learners, then in comparison to participants exposed to sentences produced with ADS, participants exposed to sentences produced with IDS would show (1) both word acceptance scores for target items and *d*’ scores above chance levels (50% and 0, respectively), (2) greater acceptance rates for target compared to filler items, and (3) greater *d*’ scores.

We also examined the use of both word length and frequency. If *ab initio* listeners more readily recognize target items with a word length more common in their native language, in our case monosyllabic words (Cutler & Carter, 1987), then we expect that in comparison to bisyllabic items, responses to monosyllabic items will show a greater difference in word acceptance rates between target and filler items as well as greater *d*’ scores. Alternatively, if an advantage for monosyllabic over bisyllabic target items is found, it could be due to the word length effect (Baddeley et al., 1975), as the shorter monosyllabic items are easier to hold in short-term memory. Finally, if frequency supports the formation of a memory trace for items in the input (Gullberg et al., 2012; Robinson, 2005), then frequently presented target items should show higher acceptance rates than infrequently presented target items when compared with chance items.

The present article was written with embedded analysis scripts in R (R Core Team, 2018) using the papaja package (Aust & Barth, 2018) in R Markdown (Allaire et al., 2018). The R Markdown and R analysis scripts as well as the de-identified data are available on Open Science Framework (https://osf.io/f2mqe/?view_only=25317ee805684313bb651dcabb37fb31). The stimuli and programs to run all three experiments are also included.

## Experiment 1

### Methods

#### Participants

Participants were recruited from the University of Maryland—College Park. Half of the participants (*n* = 21; *M* age = 20.81 years, *SD* age = 3.27; range 18–34, 13 women) were exposed to sentences produced with infant-directed speech (IDS) and the other half of the participants were exposed to sentences produced with adult-directed speech (ADS; *n* = 22; *M* age = 20.64 years, *SD* age = 1.92; range 18–26, 13 women). These sample sizes were chosen for their similarity to the sample sizes of (Gullberg et al., 2012), which had a similar experimental design in terms of between- and within-subjects manipulations. Before the experiment, each participant filled out a language use and background questionnaire to ensure that American English was the participant’s native language and that they had not had previous exposure to German or another West Germanic language besides English (e.g. Dutch, Afrikaans). All participants in the final sample had normal hearing and normal or corrected vision. An additional 6 participants were tested but not included in the final sample because they didn’t complete the experiment (*n* = 1), reported uncorrected vision impairment (*n* = 1), were not a native speaker of English (*n* = 2), or had learned German (*n* = 2). All participants were paid a small amount of money for their participation.

#### Stimuli

Following the design of Gullberg and colleagues (Gullberg et al., 2012), participants heard sentences during the exposure phase, but individual words during the test phase. Ninety-six real German words were selected to serve as target (*n* = 24; 12 monosyllabic, 12 bisyllabic) and filler (*n* = 72; 36 monosyllabic, 36 bisyllabic) items for the test phase of this study (see Appendix A1 for a list of all target and filler items). All bisyllabic items were stress-initial. No syllable was used more than once across test items, ensuring that the target and filler items were maximally different from one another. Although these target and filler items were themselves phonotactically legal in English and contained sounds with phonetic features similar to English phonemes, the sentences in which the target items were presented contained some non-English phonetic features, such as the vowels /ʏ/, /o:/, /e:/, and /a/ and the affricates /ts/ and /pf/.

The 24 target items were used to create sentences for the exposure phase. For each target item, a set of eight grammatically correct and semantically meaningful standard German sentences were created (see Appendix A2 for sentence lists), resulting in a total of 192 sentences. The target item was never placed in the initial or final position within the sentences, as utterance boundaries may facilitate recognition (Shoemaker & Rast, 2013). To control for arbitrary listening preferences and modulate the frequency of occurrence of target items during the exposure phase, two sets of sentences using a different combination of 120 sentences were created (training sets A and B). For example, in training set A, half of the target items (6 monosyllabic, 6 bisyllabic) appeared frequently (8 times each, making up 96 of the 120 trials in the exposure phase) while the other half of the target items (6 monosyllabic, 6 bisyllabic) appeared infrequently (2 times each, for 24 of the 120 trials in the exposure phase). Training set B had the same structure, but the frequent and infrequent target items were switched. For example, if *Rindvieh* was a frequent item in training set A, appearing 8 times, it would be an infrequent item in training set B, appearing 2 times. Each sentence had on average 10.41 syllables (*SD* = 2.05; range 5–15). Neither filler items nor their constituting syllables ever occurred in the sentences. Whether a participant heard training set A or B was counterbalanced across participants and registers (IDS/ADS).

Artificial language studies often use a handful of syllables in combination with one another to form words while controlling for their statistical probability. The current study used a natural language, with 441 and 445 unique syllables occurring in training sets A and B respectively. To ensure that the syllables making up the target items did not vary drastically in their transitional probability (TP) in the training sets, the TP of target item syllables was controlled for. For bisyllabic target items, the internal TP was always 1.0. The first syllable was always followed by the second syllable, and both syllables never occurred anywhere else in the sentences. For frequent mono- and bi-syllabic target items, the forward and backward TPs were 0.25 or less. This means that frequent target items could be preceded or followed by the same word no more than two times across sentences. For infrequent mono- and bi-syllabic target items, the forward and backward TPs were 0.5. Infrequent target items only appeared twice and were always preceded and followed by different words across sentences. A summary of the TPs of the target items in both training sets can be found in Appendix B.

A female, native speaker of German produced the training sentences several times in random order in both IDS and ADS. Due to the nature of IDS and ADS, the IDS recordings were longer and louder than the ADS. Similar to Thiessen et al. (2005), we addressed these possible confounds by adjusting the length and amplitude of the sentences. For each sentence, we calculated the duration of the IDS and ADS versions as well as the average of the two. Next, the duration of the IDS and ADS versions of the sentence were adjusted using the “stretch” algorithm in Adobe Audition (Adobe System Corporation, San Jose, CA) to this average. The resulting IDS and ADS sentences were therefore matched in duration (*M* = 2.83 ms; *SD* = 0.47) and speech rate (*M* = 3.68 syllables/sec; *SD* = 3.68) for the training sentences. Note that this controls for overall speech rate, but does not adjust for the exaggerated sentence- and word-final lengthening of IDS (Albin & Echols, 1996). Using Praat (Boersma & Weenink, 2016), the training sentences produced in both IDS and ADS were adjusted to the same average intensity in dB SPL. In addition, the same speaker recorded each of the 24 target items as well as 72 filler items in isolation for a total of 96 test items. These recordings were also adjusted to the same average intensity level in dB SPL.

The speaker’s average F0 was measured for each sentence. Average F0 in Hertz was significantly higher for the IDS sentences (264.86 Hz, *SD* = 20.94, 338.98 mel) compared with the ADS sentences (171.15 Hz, *SD* = 23.14, 227.93 mel), *t*(191) = 36.69, *p* < .001, *d* = 2.65. The speaker’s average minimum and maximum F0 values in Hertz for the IDS sentences (Minimum: 137.58 Hz, 185.97 mel; Maximum: 525.83 Hz, 609.59 mel) were also significantly higher than the average minimum and maximum F0 values for the ADS sentences (Minimum: 94.26 Hz, 129.96 mel; Maximum: 352.64 Hz, 435.78 mel), respectively, *t*(191) = 16.94, *p* < .001, *d* = 1.22 and *t*(191) = 14.05, *p* < .001, *d* = 1.01. These values are consistent with F0 characteristics of IDS and ADS speech in German, as reported by previous studies (Fernald et al., 1989; Schreiner et al., 2016). For the target items produced in isolation, the speaker was instructed to use a register between that of IDS and ADS, in order to reduce the likelihood that a match in register between the stimuli used at exposure and test could explain any differences in responses between the IDS and ADS registers. The speakers’ average F0 for the target items was 239.31 Hz (*SD* = 49.50, range average = 157.76–361.80; *M* mel = 309.54, range = 211.34–445.54 mel). Regarding pitch values, the target items in the test phase were more similar to the IDS exposure sentences in average and minimum F0, but more similar to the ADS exposure sentences in maximum F0.

#### Design and procedure

The experiment was presented on a 2017 iMac computer using the Open Sesame software (Mathôt et al., 2012). The experiment began with participants seated in front of a computer wearing headphones. They were instructed to simply listen to the sentences. Then, the exposure phase began and the 120 sentences were presented randomly, with the constraint that sentences containing the same target item could not occur within 5 sentences of one another. Presentation of the exposure phase lasted about 6 minutes.

After completing the exposure phase, instructions appeared on the screen, telling participants that they would be played an audio file and then would be prompted to indicate whether they had heard the sound in the exposure phase or not by pressing the corresponding key (key assignment was counterbalanced across participants). After these instructions, the test phase began. Target and filler items were presented randomly, one time each, with the constraint that items of each type (target/filler) could not be presented more than 2 times in a row. Once participants indicated their response, a fixation cross appeared on the screen for 1,000 ms, indicating that the trial had been completed. The correct expected response for all 24 target items was “present” for both training sets A and B and the correct expected response for all 72 filler items was “not-present”.

#### Data analysis

Participant responses were analyzed using R (R Core Team, 2018). Analyses of word acceptance (coded as 1 for a “present” response and 0 for a “not present” response) were modeled using mixed-effect logistic regression models (Bates et al., 2015). We used the “order” command in the buildmer package (Voeten, 2023) to determine the maximal random effects structure, including random slopes for the random intercepts of participant and item. All contrasts for fixed effects were deviation coded (−1, 1), with the exception of Frequency Status (see below). The emmeans package (Lenth, 2019) was used to report estimated marginal means for significant effects and interactions and to test post-hoc comparisons. An initial model was conducted with the intercept removed to compare target item performance against chance.

We examined the influence of Frequency Status (Frequent Target, Infrequent Target, Filler), Register (IDS, ADS), and Word Length (monosyllabic, bisyllabic) on participants’ word acceptance scores. The factor of Frequency Status reflected target items that had appeared frequently (Frequent Target) and infrequently (Infrequent Target) in the exposure phase, as well as filler items that did not appear in the exposure phase. It was deviation coded such that frequently and infrequently presented target items were separately compared to filler items. In the case of an effect of or interaction with Frequency Status, differences in word acceptance responses to frequently and infrequently presented targets were established using post-hoc comparisons of the estimated marginal means.

Analyses of *d*’ examined participants’ sensitivity to discriminate between target and filler items based on Signal Detection Theory (Stanislaw & Todorov, 1999). For each participant, we calculated the number of hits (correctly identifying a target item as “present”), correct rejections (correctly identifying a filler item as “not present”), misses (incorrectly identifying a target item as “not present”), and false alarms (incorrectly identifying a filler item as “present”). We used the psych package (Revelle, 2023) in R (R Core Team, 2018) to calculate *d*’ separately for frequently and infrequently presented target items, but using the same filler items for correct rejections and false alarms. Analyses of *d*’ were modeled using mixed-effect linear regression models (Bates et al., 2015). As in the analysis of word acceptance, we used the “order” command in the buildmer package (Voeten, 2023) to determine the maximal random effects structure, with the exception that no random intercepts for item were included as these scores cannot be calculated at the item level. All contrasts for fixed effects were deviation coded (−1, 1) and the emmeans package (Lenth, 2019) was used to report estimated marginal means for significant effects and interactions and to test post-hoc comparisons. An initial model was conducted with the intercept removed to compare *d*’ against chance.

To ensure responses did not differ between the two training sets, we compared word acceptance for target and filler items and *d*’ scores between the two training sets (training sets A, B). A general mixed-effects model of word acceptance with an interaction between Frequency Status (Frequent Target, Infrequent Target, Filler) and Training Set (A, B) had a maximum random effects structure of random intercepts for subjects and items. The main effect of Training Set (*β* = 0.01, SE = 0.1, *Z* value = 0.07, *p* = 0.95) and the interaction between Training Set and Frequency status (Frequent Targets: *β* = 0.01, SE = 0.13, *Z* value = 0.08, *p* = 0.93; Infrequent Targets: *β* = 0.02, SE = 0.13, *Z* value = 0.13, *p* = 0.9) were not significant. A t-test comparing *d*’ for participants exposed to the two training sets (A and B) revealed no significant difference, *t*(40.91) = 0.41, *p* = .682. Subsequent analyses were collapsed across training set.

### Results

#### Analyses of word acceptance

**Figure 1.**
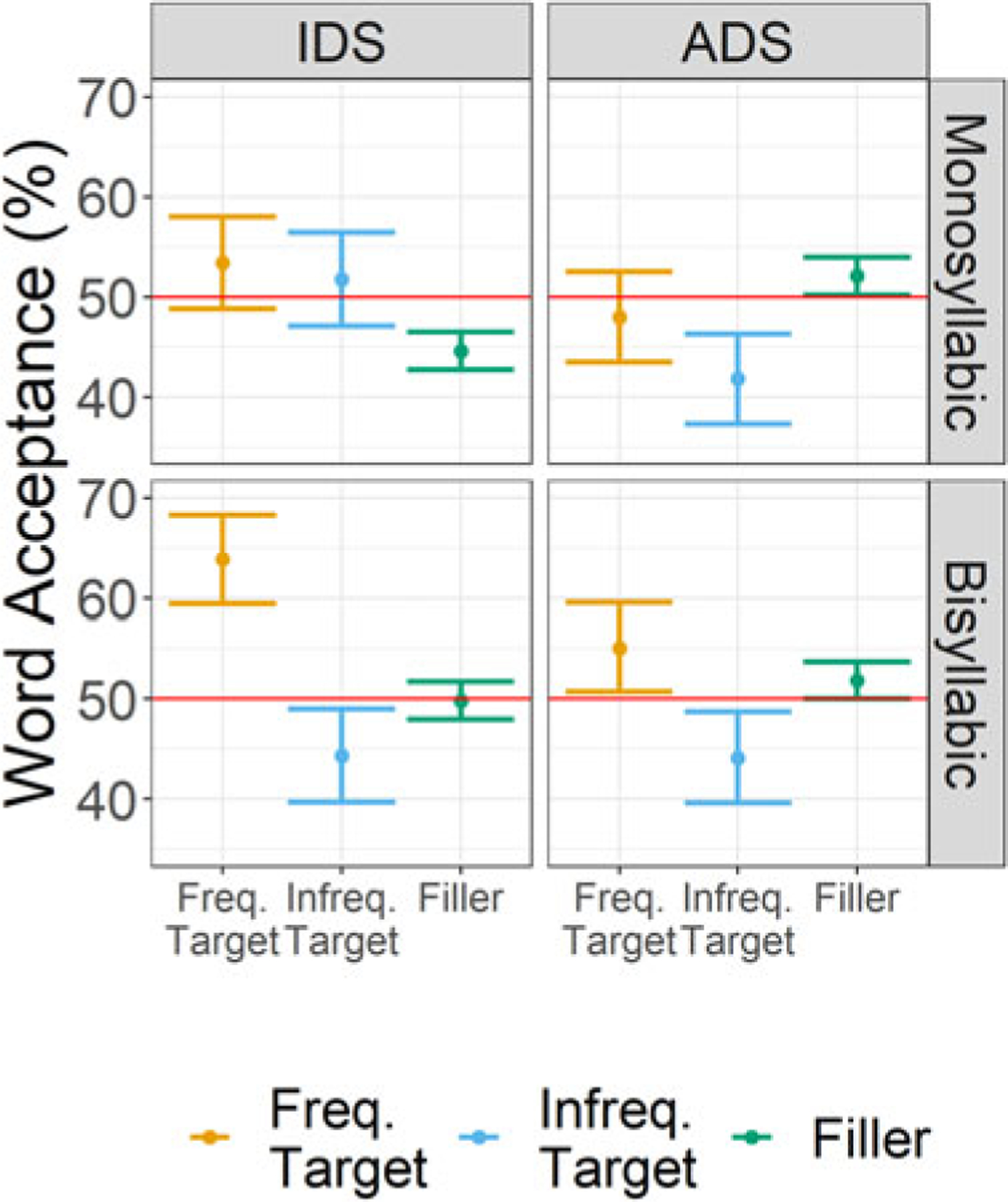
Mean word acceptance scores (%) with *+*/−1 standard error in Experiment 1 for frequently and infrequently presented target items and filler items. Responses to monosyllabic and bisyllabic test items are given in the top and bottom panels, respectively. Responses for participants trained in IDS are given in the left panels, while responses for those trained in ADS are given in the right panels. The red line at 50% indicates the chance level.

Table 1 and Figure 1 display the descriptive statistics for word acceptance for the factors of Frequency Status (Frequent Target, Infrequent Target, Filler), Register (IDS, ADS), and Word Length (monosyllabic, bisyllabic). For the comparisons of target item word acceptance to chance, the full output of the model without an intercept is given in Appendix C1. The maximum random effects structure had random intercepts for subjects and items, with a random slope for Word Length on the subjects intercept and Register on the items intercept. Word acceptance was significantly above chance for frequent, bisyllabic target items when participants were exposed to sentences produced with IDS (*β* = 0.63, SE = 0.28, *Z* value = 2.25, *p* = 0.02). All other comparisons to chance were not significant.

The general mixed-effects model of target item word acceptance with an interaction between Frequency Status (Frequent Target, Infrequent Target, Filler), Register (IDS, ADS) and Word Length (Mono-, Bisyllabic) had a maximum random effects structure of random intercepts for subjects and items, with random slopes for Register and Word Length on the subjects intercept. The comparison to filler items was significant for both Frequent Targets (*β* = 0.22, SE = 0.08, *Z* value = 2.57, *p* = 0.01) and Infrequent Targets (*β* = −0.22, SE = 0.08, *Z* value = −2.59, *p* < .01). In comparison to filler items (EMM = 0.04, SE = 0.12), acceptance of target items was greater when they were presented frequently in the exposure phase (EMM = 0.25, SE = 0.17), but smaller when they were presented infrequently (EMM = −0.18, SE = 0.17). No other main effects or interactions were significant (see Appendix C2 for full model details).

#### Analyses of d’

Table 2 displays the descriptive statistics for *d*’ for the factors of Word Length (monosyllabic, bisyllabic), Frequency (frequent, infrequent), and Register (IDS, ADS). As can be seen in Table 2, the values of *beta* were around 1.0, an indication that participants were not biased overall in their responses to accept or reject items (Hautus et al., 2021). For the comparisons of *d*’ to chance, the full output of the linear mixed-effects model without an intercept is given in Appendix C3. The maximum random effects structure had a random intercept for subjects, with a random slope for Word Length. *D*’ scores were significantly above chance for frequent, bisyllabic target items when participants were exposed to sentences produced with IDS (*β* = 0.27, SE = 0.11, *t* value = 2.45, *p* = 0.02). All other comparisons to chance were not significant.

The linear mixed-effects model of *d*’ scores with an interaction between Frequency (Frequent, Infrequent), Register (IDS, ADS) and Word Length (Mono-, Bisyllabic) had a maximum random effects structure of a random intercept for subjects, with a random slope for Word Length. The effect of Frequency was significant (*β* = 0.11, SE = 0.04, *t* value = 3, *p* < .01), showing that *d*’ scores were higher for frequently presented (EMM = 0.09, SE = 0.06) compared with infrequently presented target items (EMM = −0.12, SE = 0.06). No other main effects or interactions were significant (see Appendix C4 for full model details).

### Discussion

The results of Experiment 1 revealed evidence that L1 American-English speakers without any prior exposure to German were able to recognize words after only brief exposure to this language. Yet this was clearly a difficult task. Perhaps as a result, this finding was restricted to target items heard frequently (8 times) during the exposure phase, and strongest for bisyllabic items and in participants that were exposed to sentences produced with IDS during the exposure phase. This tentatively confirms our predictions that IDS and frequent presentation of target items during exposure aids initial word recognition in *ab initio* learners. Evidence of recognition for bisyllabic but not monosyllabic items runs counter to our predictions that *ab initio* learners should more readily segment words with a word length more common in their native language.

Strong conclusions about recognition in Experiment 1 should be interpreted with caution. Although the analysis of word acceptance suggests that participants reliably differentiated between filler items and target items that had been presented previously in the exposure phase, *d*’ scores were relatively close to 0, suggesting that participants had difficulty discriminating between target and filler items, regardless of whether they were trained using IDS or ADS or whether those items were monosyllabic or bisyllabic. Thus, while the limited effect found in the chance data is suggestive, more evidence is needed to accept these results fully.

The paradigm used in Experiment 1 required participants to listen to speech in an unknown language and hold memory traces of heard syllable strings for several minutes. Without additional cues, such as visual aids and gestural highlighting used by Gullberg et al. (2012), this may tax listeners’ working memory abilities. In Experiment 2, we examine whether *ab initio* learners are able to recognize words previously presented in our training sentences when the burden on their working memory is removed. We use the paradigm employed by Shoemaker and Rast (2013) where presentation of a sentence is immediately followed by presentation of a test item that either was or was not in the preceding sentence. In this paradigm, participants are immediately prompted to determine whether the test item was present in the sentence or not. If participants’ low performance in Experiment 1 was due to the sentence stimuli, either the stimuli items used or the recordings themselves, we should not find successful recognition when testing it using a different paradigm, and should find similar low performance using a different paradigm in Experiment 2. If their low performance is the result of the high memory demands, we should find higher performance overall in Experiment 2, but the same pattern of results. Such a replication of the basic pattern would strengthen the arguments from this current study.

## Experiment 2

### Methods

#### Participants

Sixty-three participants (*M* age = 35.19 years, *SD* age = 10.54; range 19–57, 33 women) were recruited from Prolific and tested online. This change in procedure was due to the COVID-19 pandemic. After the experiment, each participant filled out a language use and background questionnaire and completed the English and German versions of LexTALE (Lemhöfer & Broersma, 2012). This was done to ensure that American English was the participant’s native language and that they had not had previous exposure to German or another West Germanic language besides English (e.g. Dutch, Afrikaans). All participants in the final sample had normal hearing and normal or corrected vision. An additional 13 participants were tested but not included in the final sample because they did not complete the background questionnaire (*n* = 1), were not a native speaker of English (*n* = 1), had learned German (*n* = 3) or another West Germanic language (Dutch, *n* = 1), failed the attention check described below (*n* = 1), or had a technical problem while participating in the study (*n* = 6). All participants were paid a small amount of money for their participation.

#### Stimuli

To ensure counterbalancing across target and filler items as well as word length, a subset of 18 target items used in Experiment 1 were selected to serve as target items (9 monosyllabic, 9 bisyllabic). All 72 filler items from Experiment 1 also served as filler items (36 monosyllabic, 36 bisyllabic). The corresponding set of 8 sentences for each of the chosen target items from the exposure phase in Experiment 1 served as the exposure sentences in Experiment 2, for a total of 144 trials. Audio files were the same.wav files used in Experiment 1. Within each set of 8 sentences, half of the sentences were paired with the target items, and the other half were paired with one of 4 yoked filler items. Likewise, half of the sentences were produced in IDS, while the other half were produced in ADS. For each participant, 72 trials consisted of an exposure sentence produced in IDS that was either paired with the target item that appeared in the exposure sentence (*n* = 36) or a filler item that did not (*n* = 36), while the other 72 trials were produced in ADS, with the same number of pairings with target and filler items. The order of sentence register (IDS, ADS) and Word Status (Target, Filler), were counterbalanced across participants to produce 4 lists.

#### German LexTALE

Participants also completed the German version of the LexTALE task (Lemhöfer & Broersma, 2012). Participants’ averaged % correct scores were rather low (*M* = 53.12, *SD* = 6.44, range = 33.75–72.50), further supporting their responses in the language background questionnaire that they did not have any knowledge of German.

#### Design and procedure

The experiment was created using the OS Web software (Mathôt & March, n.d.) and hosted on the MindProbeEU server of JATOS (Lange et al., 2015). Participants were recruited using the platform Prolific (www.prolific.co) and were instructed to complete the study using a computer that was equipped with audio and a keyboard. To ensure that participants had working audio during the study, a catch trial was included at the beginning of the experiment that required participants to press a corresponding button based on an auditory prompt. After this audio test, they were instructed to listen to the sentence and the subsequently presented test item in isolation and their task was to decide as quickly as possible whether the isolated test item occurred in the sentence or not, pressing one button if it was heard and another button if it was not heard (“a” and “l,” counterbalanced across participants). The program waited 4,000 ms for a response before moving on. The order of trials was randomized. The interstimulus interval (ISI) between sentence and word item was 300 ms with a 100 ms jitter. Participants completed 4 practice trials before proceeding to the main experiment. During the main experiment, to maintain motivation, participants received feedback on their accuracy every 18 trials. The correct expected response for all 72 target items was for the participants to indicate they had heard the item in the sentence and the correct expected response for all 72 filler items was for the participants to indicate they had not heard the item in the sentence. After completing the main experiment, participants completed the German LexTALE task and then filled out a language background questionnaire collected using formR (Arslan et al., 2020).

#### Data analysis

The approach to data analysis was similar to Experiment 1, but with the factor of Frequency Status or Frequency removed from the analyses of word acceptance and *d*’ respectively, as Frequency was not a factor in Experiment 2. Instead, the analyses of word acceptance include the factor of Word Status (Target, Filler).

### Results

#### Analyses of word acceptance

**Figure 2.**
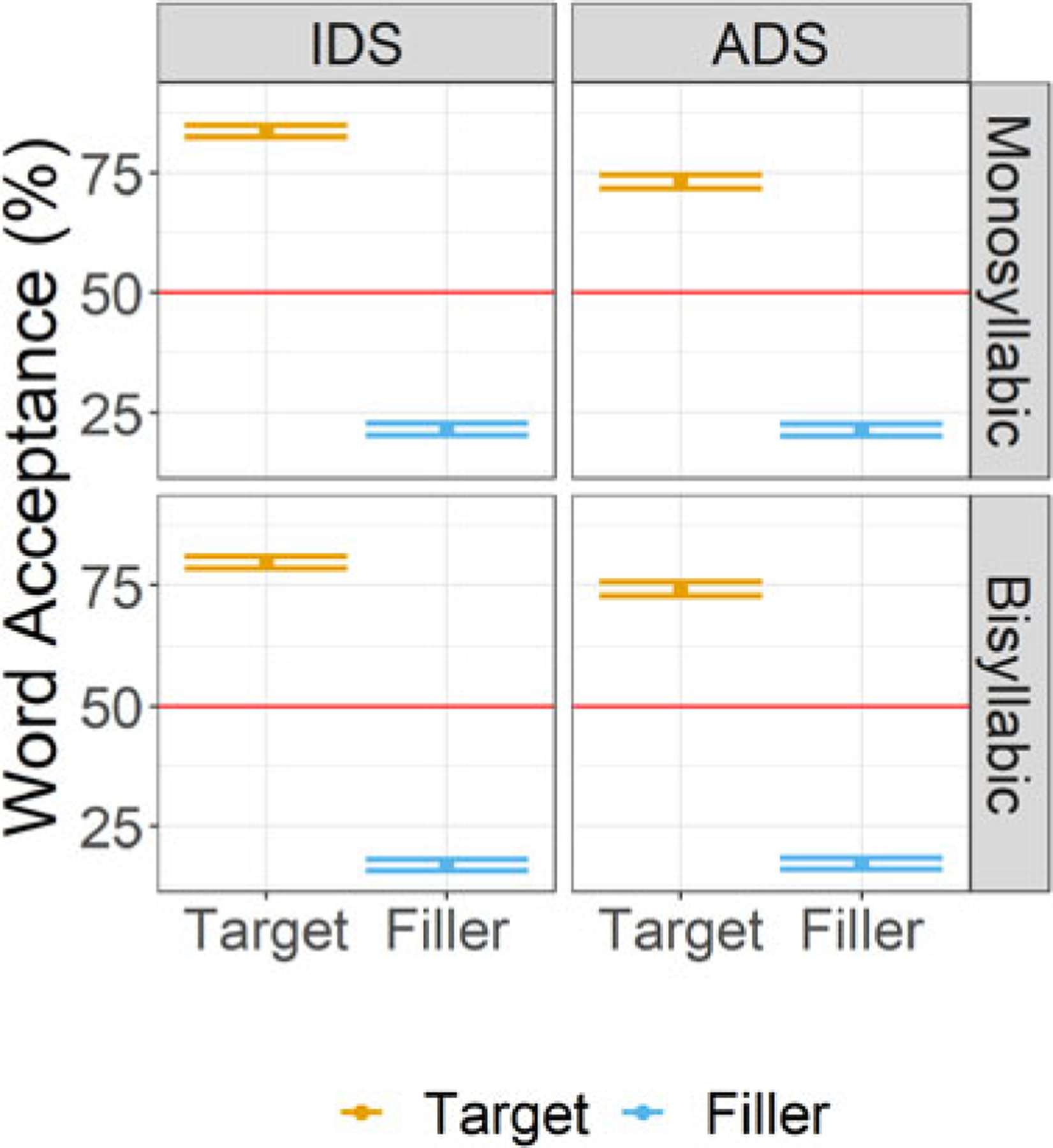
Mean word acceptance scores (%) with *+*/−1 standard error for monosyllabic and bisyllabic target and filler words following in sentences presented in IDS or ADS in Experiment 2. The red line at 50% indicates the chance level.

Table 3 and Figure 2 display the descriptive statistics for word acceptance for the factors of Word Status (target, filler), Register (IDS, ADS), and Word Length (Mono-, Bisyllabic). For the comparisons of target word acceptance to chance, the full output of the model without an intercept is given in Appendix D1. The maximum random effects structure had random intercepts for subjects and items, with random slopes for Register on both the subjects and items intercepts. Word acceptance was significantly above chance for all conditions (IDS, bisyllabic: *β* = 1.61, SE = 0.23, *Z* value = 6.87, *p* < .001; IDS, monosyllabic: *β* = 1.89, SE = 0.24, *Z* value = 8, *p* < .001; ADS, bisyllabic: *β* = 1.25, SE = 0.28, *Z* value = 4.51, *p* < .001; ADS, monosyllabic: *β* = 1.19, SE = 0.28, *Z* value = 4.29, *p* < .001).

The general mixed-effects model of target item word acceptance with an interaction between Word Status (Target, Filler), Register (IDS, ADS), and Word Length (Mono-, Bisyllabic) had a maximum random effects structure of random intercepts for subjects and items, with random slopes for Word Status, Register, and Word Length on the subjects intercept and Word Status and Register in interaction on the items intercept. The effect of Register was significant (*β* = 0.12, SE = 0.04, *Z* value = 2.82, *p* < .01), showing greater word acceptance of items following sentences that were produced in IDS (EMM = 0.05, SE = 0.1) compared with sentences produced in ADS (EMM = −0.2, SE = 0.13). The effect of Word Status was significant (*β* = 1.55, SE = 0.12, *Z* value = 12.58, *p* < .001), showing greater word acceptance for target (EMM = 1.47, SE = 0.18) compared with filler items (EMM = −1.62, SE = 0.14). The interaction between Register and Word Status was also significant (*β* = 0.11, SE = 0.04, *Z* value = 3.13, *p* < .01), showing greater acceptance of target items following sentences produced in IDS (EMM = 1.71, SE = 0.17) compared with those following sentences produced in ADS (EMM = 1.24, SE = 0.21), *β* = 0.47, SE = 0.11, *Z* value = 4.2, *p* < .001, but no difference in word acceptance for filler items (IDS: EMM = −1.61, SE = 0.15; ADS: EMM = −1.63, SE = 0.15), *β* = 0.02, SE = 0.11, *Z* value = 0.18, *p* = 0.86. All other main effects and interactions were not significant (see Appendix D2 for full model output).

#### Analysis of d’

Table 4 displays the descriptive statistics for *d*’ for the factors of Word Length (monosyllabic, bisyllabic) and Register (IDS, ADS). As can be seen in Table 4, the values of *beta* were around 1.0 or higher, an indication that if participants were biased overall in their responses to accept or reject items, they were more conservative in their responses and had higher hit and false alarm rates than rejection rates (Hautus et al., 2021). For the comparisons of *d*’ to chance, the full output of the model without an intercept is given in Appendix D3. The maximum random effects structure had a random intercept for subjects. *D*’ scores were significantly above chance for all conditions (IDS, bisyllabic: *β* = 1.85, SE = 0.08, *t* value = 22.09, *p* < .001; IDS, monosyllabic: *β* = 1.8, SE = 0.08, *t* value = 21.45, *p* < .001; ADS, bisyllabic: *β* = 1.66, SE = 0.08, *t* value = 19.88, *p* < .001; ADS, monosyllabic: *β* = 1.45, SE = 0.08, *t* value = 17.33, *p* < .001).

The linear mixed-effects model of *d*’ scores with an interaction between Register (IDS, ADS) and Word Length (Mono-, Bisyllabic) had a maximum random effects structure of a random intercept for subjects. The effect of Register was significant (*β* = 0.13, SE = 0.03, *t* value = 4.51, *p* < .001), showing that *d*’ scores were higher in response to sentences produced in IDS (EMM = 1.82, SE = 0.07) compared with ADS (EMM = 1.56, SE = 0.07). The effect of Word Length was significant (*β* = 0.07, SE = 0.03, *t* value = 2.26, *p* = 0.02), showing that *d*’ scores were higher in response to bisyllabic (EMM = 1.76, SE = 0.07) compared with monosyllabic items (EMM = 1.62, SE = 0.07). The interaction between Register and Word Length was not significant (see Appendix D4 for full model details).

### Discussion

Unlike Experiment 1, the results of Experiment 2 revealed clear evidence that L1 American English speakers, without any prior exposure to German before their participation in the experiment, were able to recognize words from German speech when this recognition was immediately tested. The significant effect of Word Status and high participant *d*’ scores suggest successful discrimination of target and filler items. If participants’ lack of success in Experiment 1 was due to peculiarities of the sentence stimuli, either the stimulus items used or the recordings themselves, we would not have found successful word recognition using a different paradigm. This suggests that the low performance in Experiment 1 may be due to the requirement of the paradigm, specifically that participants needed to hold memory traces of several heard syllable strings simultaneously for several minutes. In contrast, participants in Experiment 2 only needed to hold the memory trace of one syllable string for a few seconds.

Experiment 2 also revealed a clear advantage in word recognition for bisyllabic compared with monosyllabic items. As in Experiment 1, this again suggests that the bisyllabic advantage found by Gullberg and colleagues (Gullberg et al., 2012) was not necessarily due to word length frequency in the first language, as monosyllabic words are more frequent in English (Cutler & Carter, 1987). Rather, there appears to be something about bisyllabic items that makes them easier to recognize from fluent speech than monosyllabic items regardless of their frequency in the particular language.

Participants also had a higher accuracy and greater discrimination for IDS compared with ADS trials. IDS has likewise been found to facilitate speech segmentation in infants (Singh et al., 2009; Thiessen et al., 2005) and word learning in adult *ab initio* learners (Golinkoff & Alioto, 1995). The latter study with adult *ab initio* learners could not pinpoint at what point IDS facilitated the word learning process, as it could have occurred during exposure to the continuous speech, label-referent association, or during both. The results of Experiment 2, which included no label-referent association task and was entirely auditory, suggests that IDS is facilitative at the level of processing and storing continuous speech in adult *ab initio* learners. This may have also been the case in Experiment 1, but by using a paradigm that reduced memory demands in Experiment 2 we were able to detect this difference.

Experiments 1 and 2 also differed in the population of participants recruited as well as the settings where participants were tested: university students and in-lab for Experiment 1 and general population and remote for Experiment 2. The participants recruited to participate remotely using Prolific in Experiment 2 had a wide range of educational backgrounds and were considerably older (*M* = 35.19) than the undergraduate students recruited in Experiment 1 (20.72), following the typical pattern of a more diverse subject pool for remote studies (Paolacci et al., 2010). Yet the pattern of results would likely be reversed if they were due to differences in participant age, as younger adults have higher working memory skills (Linares et al., 2016). Furthermore, the setting itself is not likely to play a large role. Using an exposure-test paradigm similar to that of Experiment 1, Hartshorne et al. (2019) replicated evidence of statistical word segmentation from several previous studies with a participant population tested in a remote setting. To ensure that the more successful word recognition found in Experiment 2 compared to Experiment 1 was due to the difference in paradigm and not due to a setting change, Experiment 3 tests the paradigm used in Experiment 1, but with participants recruited online using the platform Prolific, as in Experiment 2.

## Experiment 3

### Methods

#### Participants

Participants were recruited from Prolific and tested online. Half of the participants (*n* = 21; *M* age = 34.14 years, *SD* age = 9.17; range 21−55, 14 women) were exposed to sentences produced with infant-directed speech (IDS) and the other half of the participants were exposed to sentences produced with adult-directed speech (ADS; *n* = 23; *M* age = 39.91 years, *SD* age = 11.32; range 21−59, 12 women). Similar to Experiment 1, these sample sizes were chosen for their similarity to the sample sizes of (Gullberg et al., 2012), which had a similar experimental design in terms of between- and within-subjects manipulations. After the experiment, each participant filled out a language use and background questionnaire and completed the English and German versions of LexTALE (Lemhöfer & Broersma, 2012). This was done to ensure that American English was the participant’s native language and that they had not had previous exposure to German or another West Germanic language besides English (e.g. Dutch, Afrikaans). All participants in the final sample had normal hearing and normal or corrected vision. An additional 4 participants were tested but not included in the final sample because they failed the attention check (*n* = 2), or had a technical problem while participating in the study (*n* = 2). All participants were paid a small amount of money for their participation.

#### Stimuli

The stimuli were identical to the stimuli used in Experiment 1.

#### German LexTALE

Participants also completed the German version of the LexTALE task (Lemhöfer & Broersma, 2012). Participants’ averaged % correct scores were rather low (*M* = 53.61, *SD* = 4.94, range = 41.25−65), further supporting their responses in the language background questionnaire that they did not have any knowledge of German.

#### Design and procedure

Experiment 3 used the same design as Experiment 1. As in Experiment 2, the experiment was created using the OS Web software (Mathôt & March, 2022) and hosted on the MindProbeEU server of JATOS (Lange et al., 2015). Participants were recruited using the platform Prolific and were instructed to complete the study using a computer that was equipped with audio and a keyboard. To ensure that participants had working audio during the study, a catch trial was included at the beginning of the experiment that required participants to press a corresponding button based on an auditory prompt. Unlike Experiment 2, Experiment 3 required participants to listen to an exposure phase that lasted for several minutes. To ensure participants did not just start the experiment and walk away from their computer, a letter appeared for 2 seconds every 12 sentences during the exposure phase and participants were prompted to indicate which letter they saw displayed. All participants correctly identified at least 8 of the 10 letters, indicating sustained attention during the exposure phase.

#### Data analysis

The approach to data analysis was identical to Experiment 1.

To ensure that the two training sets did not lead to different patterns of results, we first examined a general mixed-effects model of word acceptance with an interaction between Frequency Status (Frequent Target, Infrequent Target, Filler) and Training Set (A, B) with a maximum random effects structure of random intercepts for subjects and items, with a random slope for Training Set on the items intercept. The main effect of Training Set (*β* = −0.13, SE = 0.1, *Z* value = −1.39, *p* = 0.17) and the interaction between Training Set and Frequency status (Frequent Targets: *β* = −0.12, SE = 0.12, *Z* value = −0.94, *p* = 0.35; Infrequent Targets: *β* = 0.18, SE = 0.12, *Z* value = 1.5, *p* = 0.13) were not significant. A t-test comparing *d*’ for the two training sets (A and B) revealed no significant difference, *t*(40.80) = 1.83, *p* = .075. Given the lack of evidence for a difference in performance between participants that were exposed to the two different training sets, all subsequent analyses are conducted on data collapsed by training set.

### Results

#### Analyses of word acceptance

**Figure 3.**
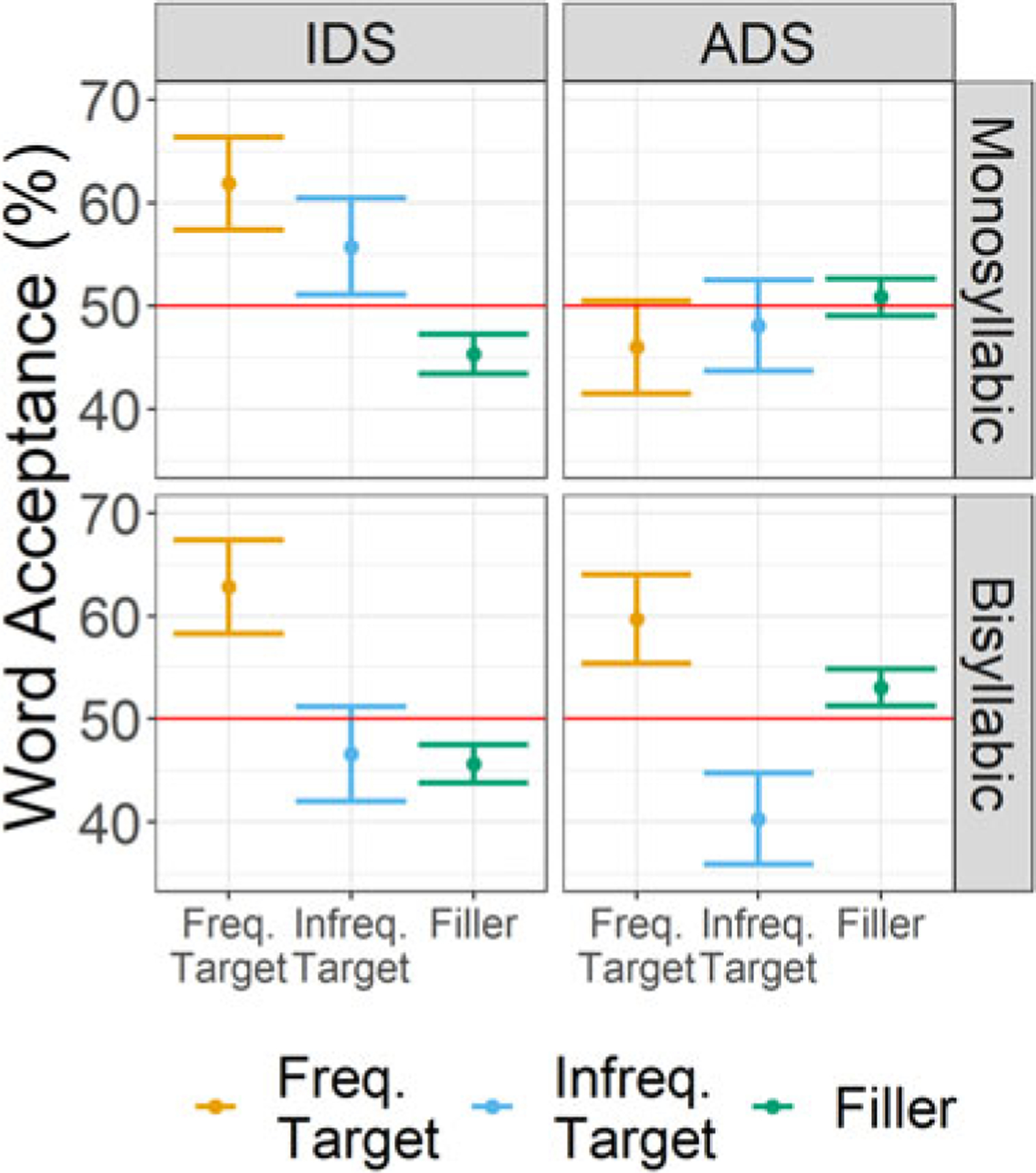
Mean word acceptance scores (%) with *+*/−1 standard error in Experiment 3 for frequently and infrequently presented target items and filler items. Responses to monosyllabic and bisyllabic test items are given in the top and bottom panels, respectively. Responses for participants trained in IDS are given in the left panels, while responses for those trained in ADS are given in the right panels. The red line at 50% indicates the chance level.

Table 5 and Figure 3 display the descriptive statistics for word acceptance for the factors of Frequency Status (Frequent Target, Infrequent Target, Filler), Register (IDS, ADS), and Word Length (monosyllabic, bisyllabic). For the comparisons of target word acceptance to chance, the full output of the model without an intercept is given in Appendix E1. The maximum random effects structure had random intercepts for subjects and items. Word acceptance was significantly above chance when participants were exposed to sentences produced with IDS for frequent, bisyllabic target items (*β* = 0.54, SE = 0.24, *Z* value = 2.21, *p* = 0.03) and frequent, monosyllabic target items (*β* = 0.49, SE = 0.24, *Z* value = 2.04, *p* = 0.04). All other comparisons to chance were not significant.

The general mixed-effects model of target item word acceptance with an interaction between Frequency Status (Frequent Target, Infrequent Target, Filler), Register (IDS, ADS) and Word Length (Mono-, Bisyllabic) had a maximum random effects structure of random intercepts for subjects and items, with a random slope for Word Length on the subjects intercept. The comparison to filler items was significant for both Frequent Targets (*β* = 0.25, SE = 0.08, *Z* value = 2.97, *p* < .01) and Infrequent Targets (*β* = −0.21, SE = 0.08, *Z* value = −2.47, *p* = 0.01). In comparison to filler items (EMM = 0.04, SE = 0.11), acceptance of target items was greater when they were presented frequently in the exposure phase (EMM = 0.34, SE = 0.17), but smaller when they were presented infrequently (EMM = −0.12, SE = 0.16). The interaction between Frequency Status and Word Length was also significant (Frequent Targets: *β* = 0.21, SE = 0.08, *Z* value = 2.44, *p* = 0.01; Infrequent Targets: *β* = −0.19, SE = 0.08, *Z* value = −2.28, *p* = 0.02). Word acceptance was significantly greater for frequently presented bisyllabic target items (EMM = 0.53, SE = 0.23) compared with infrequently presented bisyllabic target items (EMM = −0.33, SE = 0.22), *β* = 0.85, SE = 0.2, *Z* value = 4.26, *p* < .001, but no difference in word acceptance between frequently and infrequently presented monosyllabic target items (frequent, monosyllabic: EMM = 0.15, SE = 0.21; infrequent, monosyllabic: EMM = 0.09, SE = 0.21), *β* = 0.06, SE = 0.19, *Z* value = 0.31, *p* = 0.95. All other main effects and interactions were not significant (see Appendix E2 for full model output).

#### Analyses of d’

Table 6 displays the descriptive statistics for *d*’ for the factors of Word Length (monosyllabic, bisyllabic), Frequency (frequent, infrequent), and Register (IDS, ADS). As can be seen in Table 6, the values of *beta* were around 1.0, an indication that participants were not biased overall in their responses to accept or reject items (Hautus et al., 2021). For the comparisons of *d*’ to chance, the full output of the model without an intercept is given in Appendix E3. The maximum random effects structure had a random intercept for subjects, with random slopes for Word Length and Frequency. *D*’ scores were significantly above chance for frequent, bisyllabic target items when participants were exposed to sentences produced with IDS (*β* = 0.22, SE = 0.11, *t* value = 2.05, *p* = 0.04) and ADS (*β* = 0.35, SE = 0.1, *t* value = 3.39, *p* < .01). All other comparisons to chance were not significant.

The linear mixed-effects model of *d*’ scores with an interaction between Frequency (Frequent, Infrequent), Register (IDS, ADS) and Word Length (Mono-, Bisyllabic) had a maximum random effects structure of a random intercept for subjects, with random slopes for Word Length and Frequency. The effect of Frequency was significant (*β* = 0.12, SE = 0.04, *t* value = 2.68, *p* = 0.01), showing that *d*’ scores were higher for frequently presented (EMM = 0.17, SE = 0.06) compared with infrequently presented target items (EMM = −0.07, SE = 0.07). The interaction between Frequency and Word Length was also significant, (*β* = 0.1, SE = 0.03, *t* value = 3.13, *p* < .01). *D*’ scores were higher for frequently presented bisyllabic (EMM = 0.28, SE = 0.08) compared with frequently presented monosyllabic items (EMM = 0.05, SE = 0.09, *β* = 0.24, SE = 0.11, *t* value = 2.1, *p* = 0.04) as well as higher for frequently presented compared with infrequently presented bisyllabic items (EMM = −0.16, SE = 0.09, *β* = 0.44, SE = 0.11, *t* value = 3.93, *p* < .001). No other main effects or interactions were significant (see Appendix E4 for full model details).

### Discussion

The results of Experiment 3 again reveal evidence that adult *ab initio* learners were able to recognize words after brief exposure to German speech. This pattern was restricted to items heard frequently during exposure (8 times). Furthermore, the word acceptance analysis found that only participants exposed to sentences produced with IDS recognized these items. The *d*’ analysis found that for bisyllabic target items, participants exposed to sentences produced with either IDS or ADS were able to recognize these items. Similar to Experiment 1, we again find support for our prediction that frequent presentation of target items during exposure aids initial word recognition in *ab initio* learners as well as evidence against our prediction that *ab initio* learners should more readily recognize monosyllabic items.

In regards to the role of speech register, the word acceptance analysis again found evidence for item word acceptance only in participants that had been exposed to sentences produced with IDS compared to those that had been exposed to sentences produced with ADS, similar to that of Experiment 1. Yet, the analysis of *d*’ scores did not find a similar advantage for IDS over ADS. This does not provide strong evidence that IDS speech facilitates initial word recognition in adult *ab initio* learners, at least when participants must hold the forms of newly encountered words over several minutes before their recognition is tested.

The analysis of word acceptance again suggests that participants reliably differentiated between filler items and target items that had been presented previously in the exposure phase, but as in Experiment 1, *d*’ scores were low. This again leads us to be cautious about claims of recognition. This pattern of results mirrors the results of Experiment 1, but in a new, older population tested remotely.

## General discussion

The current study investigated whether adult *ab initio* learners’ ability to recognize words after brief exposure to continuous speech in a new language is improved when that speech is produced using an infant-directed register. Across all experiments, we found that participants had a higher probability of reporting that they had previously heard a target item when they had been exposed to it embedded in speech produced in IDS. Repeated exposure to target items during the exposure phase also improved word recognition (Experiments 1 and 3) and recognition was higher for bisyllabic compared with monosyllabic items (Experiments 2 and 3, but not 1). Across experiments, evidence for word recognition was robust when task demands were lowered (Experiment 2), but less robust when task demands were higher (Experiments 1 and 3). In the following sections, we discuss our findings in regards to speech register, frequency and word length effects, and the role of task on word recognition in continuous speech at first exposure.

### Infant- and adult-directed speech

As predicted, IDS was found to facilitate word recognition in adult *ab initio* learners. This was shown most clearly in Experiment 2, where target item recognition was greater following sentences produced with IDS compared with ADS. The advantage for IDS over ADS was only borne out in the chance comparisons in Experiment 1 and to a lesser extent in Experiment 3. We return to the methodological differences between Experiment 2 and Experiments 1 and 3 later in the General Discussion. Previous research has shown that IDS facilitates segmentation in English-learning infants (Singh et al., 2009; Thiessen et al., 2005). The current study extends these results to a new learner population, showing that the exaggerated pitch and intonation in this register supports word recognition in adult *ab initio* leaners. Previously, word learning studies with adult *ab initio* learners had shown that simultaneous continuous speech processing and label-referent association is facilitated by IDS (Golinkoff & Alioto, 1995; Ma et al., 2020), but these studies were unable to pinpoint the specific role played by IDS. Our study shows that even in the absence of a word learning task, IDS is facilitatory for adult *ab initio* learners’ initial word recognition.

Infant-directed speech differs from ADS in ways that may particularly support initial word recognition in continuous speech. The slower speech rate of IDS, due to longer pauses at phrasal boundaries (Fernald & Mazzie, 1991), may aid segmentation (Gout et al., 2004). But, it is unlikely that this played a role in the current study. We equated the speech rate of our IDS and ADS stimuli, reducing the influence that speech rate may have on the results. Although this equated for overall speech rate in the IDS and ADS stimuli, variability in the duration of individual segments or syllables, especially phrase-final lengthening in IDS, could have still been present (e.g., Martin et al., 2016; White et al., 2020). The sentences used in our stimuli, however, never placed target items in the final position, limiting any role this variation could have played in the results.

IDS and ADS also differ in their pitch and intonation. Pitch is a reliable cue to stress in German (Jessen et al., 1995). Like English, German is a stress-timed language and evidence suggests that both English- and German-learning infants successfully use the dominant stress pattern, the trochaic unit, to segment words (Höhle et al., 2009; Jusczyk et al., 1999). IDS has been reported to have exaggerated prosodic features in comparison to ADS, including exaggerated pitch peaks (Fernald & Cummings, 2003), perhaps because lexical stress is more likely to be accented in IDS. Nonetheless, when the occurrence of pitch peaks is equated across word syllables, effectively removing it as a segmentation cue, an advantage for IDS is still found for infant word segmentation (Thiessen et al., 2005). The exaggerated pitch and intonation of IDS may therefore not be the entire source of the facilitation effect in word recognition. To better understand whether it was the application of segmentation strategies from English that led the participants in the current study to more easily discover word boundaries in IDS, future studies should compare word recognition performance when exaggerated pitch peaks are equated.

In addition to the modifications to speech itself, increased attention has also been proposed as the underlying mechanism for the facilitatory role that IDS seems to play in infant language acquisition (Rose, Feldman, & Jankowski, 2003). IDS appears to attract infants’ attention to the speech signal, as they have been found to attend longer to stimuli produced in IDS compared with ADS (e.g. Cooper & Aslin, 1990; The ManyBabies Consortium, 2019). Thiessen et al. (2005) proposed different hypotheses for how the increased attention to IDS may lead to an advantage for infant word segmentation. On the one hand, segmentation abilities may have been equal for infants familiarized with IDS and ADS, but infants familiarized with IDS may have improved memory for the items segmented during the exposure phase, resulting in successful discrimination between target and filler items when tested in the test phase. On the other hand, by improving attention during the exposure phase, infants may have been more likely to detect syllable co-occurrences in the speech stream, placing the advantage for IDS in segmentation on the process itself and not memory retention.

If participants were more attentive to the exposure sentences produced in IDS, this could have boosted their memory processes and improved recognition at test compared with participants that heard ADS during the exposure phase. We did not directly measure participants’ attention during the exposure phase and adults’ preferences for IDS and ADS are not well known. But, the experiments in the current study differed in their attentional and memory requirements. In comparison to Experiment 2, the exposure phases of Experiments 1 and 3 required longer sustained attention and their test phases required longer-term memory of items presented during the exposure phase. If increased attention to IDS was the driving force behind improved word recognition in our study, then we would have likely observed a greater difference in performance for participants exposed to sentences produced with IDS and ADS in Experiments 1 and 3. Instead, we found the greatest difference in performance between IDS and ADS when participants were immediately tested on their detection of the target items presented in the sentences of the exposure phase (Experiment 2). Nonetheless, the current study cannot determine whether the source of the facilitatory effect of IDS on word recognition in adult *ab initio* learners is due to increased attention.

When recording stimuli for the current study, the speaker was instructed to produce the isolated-word test items in a register intermediate to IDS and ADS so that they were not an exact match to one register or the other. However, we were not trying to ensure equivalent similarity between isolated words and the IDS vs. ADS sentences, either in terms of pitch nor in terms of vowel quality, formant measurements, or relative durations, which varied across the different productions. It is therefore possible that some test items were better matched along these dimensions. For example, the average and minimum pitch values of the target items were more similar to the IDS exposure sentences, but maximum pitch values of the target items were more similar to the ADS exposure sentences. Despite these potential discrepancies, previous evidence suggests that listeners do not seem to show strong effects of acoustic matching in L2 word learning. Instead, exposure to greater acoustic variability appears to promote word learning and recall (e.g., Barcroft & Sommers, 2005). It therefore seems unlikely that the IDS advantage in word recognition in the current study is due to matching (or greater similarity) between exposure and test items of particular acoustic characteristics, although this cannot be definitively ruled out.

Decades of research have been devoted to the study of IDS and its role in language learning, whether in adults or infants. Less is known, however, about the speech register that adult learners of a new language are more likely to receive in their input, known as foreigner-directed speech (FDS). Like IDS, FDS may have exaggerated pitch (Papoušek & Hwang, 1991; but see Biersack et al., 2005; Uther et al., 2007) or hyperarticulated vowels (Uther et al., 2007). This may suggest that the advantages found here for IDS would generalize to FDS. That said, the changes in FDS are often smaller than those made for infants (e.g., Uther et al., 2007); thus, whether the speech modifications found in FDS directly influence word recognition or language learning in general, remain untested.

### Frequency and word length effects

In addition to comparing performance when participants were familiarized with input produced in IDS or ADS, we also investigated the role of frequency and word length, two aspects of the speech input that have been previously found to influence adult *ab initio* learners’ word recognition (Gullberg et al., 2012). As predicted, items that appeared frequently (8 times) in the exposure phase were more readily recognized than infrequently presented items (2 times). These results are consistent with previous research, which has shown the presentation of eight, but not four or less, instances of a target item are enough for a memory trace to be formed for newly encountered words in an L2 (Gullberg et al., 2012; Snijders et al., 2007).

Participants were also predicted to be influenced by word length, specifically that we expected to find an advantage in recognition for monosyllabic items. Instead, we found an advantage for bisyllabic items in that sensitivity to target items (*d*’) was higher for bisyllabic compared with monosyllabic items in Experiment 2 and that only bisyllabic, but not monosyllabic items, that were presented frequently to participants exposed to sentences produced in IDS showed above chance recognition. But, in Experiment 3, participants exposed to sentences produced with IDS showed above-chance recognition for both bisyllabic and monosyllabic frequently presented target items. Previous research has also found that Dutch listeners more readily recognize bisyllabic compared with monosyllabic items from Chinese speech (Gullberg et al., 2012), which could have been due to the higher frequency of bisyllabic words in Dutch (Vroomen et al., 1996) or because the trochaic stress pattern of Mandarin Chinese bisyllabic items (Lai et al., 2010) may be a word segmentation cue for Dutch listeners (Vroomen et al., 1996). In contrast to Dutch, bisyllabic words are not more frequent than monosyllabic words in English (Cutler & Carter, 1987), making the bisyllabic advantage found in the current study unlikely to be due to the frequency patterns in English. Like Mandarin Chinese and Dutch, German and English also have an overwhelmingly trochaic stress pattern and like in Dutch, this is a word segmentation cue for English listeners (e.g., Cutler & Butterfield, 1992). Considering that monosyllabic words are also stressed in German, participants may have correctly detected the onset boundary for these items with ease, but perhaps struggled to determine where the offset boundary occurred, as we did not control for the stress pattern of words following target items in our sentences. Nonetheless, monosyllabic and bisyllabic words were similarly followed by words beginning with stressed (monosyllabic: *n* = 79; bisyllabic: *n* = 85) and unstressed syllables (monosyllabic: *n* = 17; bisyllabic: *n* = 11), making it unlikely that this influenced the pattern of results. Future studies will be needed to determine whether *ab initio* learners benefit from the prosodic similarity in word syllable structure between the target language and their first language.

Despite finding these predicted effects of frequency and word length, these effects appear to be separate from effects of IDS. Across 3 experiments, neither of frequency nor word length interacted with register. Thus, the influence of item frequency or word length may be independent from the influence of register.

### The role of task on word recognition at first exposure

When participants were required to first listen to speech in an unknown language and hold memory traces of heard syllable strings for several minutes before their word recognition was tested in Experiments 1 and 3, their overall segmentation and recognition performance was not strong, regardless of whether they were exposed to the IDS or ADS register. There was evidence for increased acceptance of target items heard in the exposure phase as previously heard items, but only for a restricted set of conditions and *d*’ scores were close to 0. This is contrary to the results of Gullberg et al. (2012), who found successful word recognition in ab-initio learners using a similar paradigm with the same number of sentences in the exposure phase and the same number of target and filler items in the test phase.

There are several differences between the study of Gullberg et al. (2012) that may have contributed to participants’ differences in success. First, the exposure phase used by Gullberg et al. (2012) was accompanied by a visual stimulus whereas the exposure phase was auditory only in the current study. Participants in Gullberg’s study viewed a weather report while they were exposed to the unknown language, which included some gestural highlighting and referents for some of the target items. Although Gullberg et al. (2012) found successful word recognition when testing specifically for word recognition and not sound-to-meaning mapping, the presence of these visual cues during the exposure phase may have improved participants’ processing of the speech. Speech shadowing is improved when participants also have access to a video of the speaker (Reisberg, McLean, & Goldfield, 1987), although visual cues do not seem to reduce the demands of listening effort (Picou & Ricketts, 2014). The weather report could have also improved participants’ attentiveness, leading to better memory formation, which would have impacted their recognition in the test phase. Although this is a possible explanation for our Experiment 1, the current Experiment 3 included catch trials, where participants needed to correctly identify letters that occasionally appeared on the screen. Although not visual cues *per se*, the addition of catch trials could have improved participant attentiveness during the exposure phase, or it could have distracted participants and interrupted their processing of the speech, as the catch trials were unrelated to the participants’ task of listening to the speech. Nonetheless, the results of Experiments 1 and 3 were similar in regards to word recognition, so the use of catch trials did not appear to impact word recognition performance differently in comparison to Experiment 1.

Second, the sentences used in the current study were created such that the target item never appeared in the initial or final position of the sentence, whereas target items sometimes appeared in the initial or final position of the sentences used by Gullberg et al. (2012). The recognition of items placed at the edges of utterances is likely easier, as the edge provides a cue to one of the item’s boundaries through silence. Word recognition is facilitated in both infant and adult *ab initio* learners for words positioned at utterance edges (Seidl & Johnson, 2006; Shoemaker & Rast, 2013). Although the sentences used by Gullberg and colleagues (Gullberg et al., 2012) did not consistently position target items at the edges, this was an additional cue that participants could have used on some items, which would have boosted their performance in their task relative to the current study.

Finally, although both Gullberg and colleagues (Gullberg et al., 2012) and the current study controlled for the frequency of appearance of target items in the exposure phase, the training sets used in the current study additionally controlled for the transitional probabilities of the syllables used in the monosyllabic and bisyllabic target items. In the sentence stimuli used by Gullberg et al. (2012), some of the syllables occurring in the target bisyllabic items also occurred elsewhere in the exposure phase, resulting in an internal TP ranging between 0.25 and 1 for bisyllabic items. The boundaries around the target items were also not controlled for, resulting in a TP between 0.067 and 1 at word boundaries. In the current study, bisyllabic items had an internal TP of 1.0, a clear statistical cue that has been found to promote syllable coherence in adults and infants (Saffran et al., 1996, 1997). Word boundaries around the target items were kept to a TP between 0.25 and 0.50, depending on the frequency of that target item’s occurrence in the exposure phase, which reduced the likelihood that listeners would consider spurious co-occurrences between target syllables and surrounding syllables to form potential words. As a result, the additional statistical cues present in the exposure phases of Experiments 1 and 3 in the current study should have resulted in improved word recognition in comparison to an exposure phase where these cues were not controlled for. Nonetheless, the results of the current study showed weaker evidence of word recognition in comparison to Gullberg et al. (2012), making it unlikely that the additional statistical cues present in the current study explain the difference in success between the two studies.

It is important to note that the current study does not demonstrate that participants *only* recognized the form they were tested on and that there is only one potential way of parsing the speech. Participants could have remembered all sequences they heard in the input, with or without correctly placed word boundaries, and accessed this information during the test phase. This would especially be the case in Experiment 2, which significantly reduced memory loads and where the full sequence of the sentence may still be in working memory at test. Take the example sentence “Der Gips befindet sich in der Treppe” (The plaster is in the stairs). This sentence contains a target item, the monosyllabic word “Gips” which is a stressed syllable in German. The stress-based segmentation strategy used in English will likely lead listeners to correctly place a word boundary at the onset of “Gips,” but more information would be needed to determine how much more of the input to include in the segmented word. “Gips” is followed by the trisyllabic word “befindet,” which has a weak-strong-weak stress pattern. The participant could have placed the coda word boundary after the weak syllable “be”, as it is followed by the strong syllable “fin”, resulting in the segmented form “Gipsbe”. As we only tested participants on their recognition of the correct forms, we are unable to determine whether this is the case, especially in Experiment 2, where participants only heard each target item once before they gave their response. Studies that examine the use of statistical regularities in the input to segment speech often compare responses to “words”, where the syllables have a high co-occurrence in the input (high transitional probability), to “partwords”, where the syllables appear with equal frequency as “words” but with a low co-occurrence in the input (low transitional probability), finding higher accuracy for “words” compared with “partwords” as well as items that did not appear in the input (e.g. Kittleson et al., 2010; Pelucchi et al., 2009). The use of “partwords”, such as “Gipsbe”, during a testing phase as test items could be an approach to determine the exact form that participants segmented from the speech. We therefore restrict our claims to that of recognition of word forms encountered in continuous speech in the current study. Nonetheless, our study does provide evidence that participants were able to find and remember (for varying lengths of time) sound information, which is certainly one of the first steps in segmentation and the acquisition of a foreign language.

Related to the previous point, the current study does not identify the exact form that participants recalled from the exposure phase in Experiments 1 and 3 or the sentences in Experiment 2. It is possible that even if participants had segmented the item “Gips”, their representation for this item is not fully specified, meaning that participants may have also accepted a phonological alteration such as “Kips” or “Goops” (e.g. Sebastián-Gallés, Echeverría, & Bosch, 2005). Furthermore, this representation may depend on the similarity of the sounds in the item and their similarity to the phonological inventory of the listener (Best et al., 2001). The target and filler items used in the current study were chosen for their English-like phonetic features, to limit the potential mismatch between our test items and participants’ native phonemic inventory. Nonetheless, the ability to represent newly encountered words and recognize them again in a different context or at a later time are essential components of foreign language acquisition. Investigation of this ability and its developmental trajectory are a promising avenue for future research.

## Conclusion

The current study investigated whether adult *ab initio* learners’ ability to recognize words after brief exposure to speech in a new language is improved when that speech is produced using an infant-directed register. The results showed that adult *ab initio* learners also benefit from infant-directed speech and were more successful when they were exposed to items more frequently in the input and at detecting bisyllabic compared with monosyllabic items. Nonetheless, participants’ recognition performance was lower than in previous studies, which may be due to the auditory-only nature of the current study or the position of the target items within the sentences used in the exposure phase.

## Supplementary Material

1

**Supplementary material.** The supplementary material for this article can be found at https://doi.org/10.1017/S0142716425100350

## Figures and Tables

**Table 1.** Means, standard deviations, and 95% confidence intervals for word acceptance rates for monosyllabic and bisyllabic target items, presented frequently and infrequently, as well as filler items for participants exposed to sentences produced with IDS or ADS in Experiment 1

Word length	Frequency status	IDS			ADS		
		Mean	SD	CI	Mean	SD	CI
Monosyllabic	Frequent target	0.534	0.501	[0.443, 0.625]	0.48	0.502	[0.39, 0.57]
	Infrequent target	0.517	0.502	[0.425, 0.609]	0.418	0.495	[0.329, 0.507]
	Filler	0.554	0.497	[0.517, 0.591]	0.479	0.5	[0.443, 0.515]
Bisyllabic	Frequent target	0.639	0.482	[0.551, 0.727]	0.551	0.499	[0.463, 0.639]
	Infrequent target	0.443	0.499	[0.351, 0.535]	0.442	0.499	[0.352, 0.532]
	Filler	0.501	0.5	[0.464, 0.538]	0.481	0.5	[0.445, 0.517]

**Table 2.** Means, standard deviations, and 95% confidence intervals for d’ and beta for bisyllabic and monosyllabic items presented frequently and infrequently to participants exposed to sentences produced with IDS or ADS in Experiment 1

Measure	Syllable	Frequency	IDS			ADS		
			Mean	SD	CI	Mean	SD	CI
*d*’	Monosyllabic	Frequent target	−0.076	0.493	[−0.3, 0.148]	0.002	0.593	[−0.261, 0.265]
		Infrequent target	−0.083	0.627	[−0.368, 0.202]	−0.161	0.659	[−0.453, 0.131]
	Bisyllabic	Frequent target	0.268	0.513	[0.034, 0.502]	0.155	0.459	[−0.048, 0.358]
		Infrequent target	−0.161	0.531	[−0.403, 0.081]	−0.092	0.472	[−0.301, 0.117]
beta	Monosyllabic	Frequent target	1.015	0.227	[0.912, 1.118]	0.908	0.17	[0.833, 0.983]
		Infrequent target	0.945	0.337	[0.792, 1.098]	0.843	0.242	[0.736, 0.95]
	Bisyllabic	Frequent target	1.29	1.551	[0.584, 1.996]	0.93	0.149	[0.864, 0.996]
		Infrequent target	1.095	0.732	[0.762, 1.428]	0.907	0.176	[0.829, 0.985]

**Table 3.** Means, standard deviations, and 95% confidence intervals for word acceptance rates for monosyllabic and bisyllabic target and filler items for participants exposed to sentences produced with IDS or ADS in Experiment 2

Word length	Word status	IDS			ADS		
		Mean	SD	CI	Mean	SD	CI
Monosyllabic	Target	0.836	0.37	[0.813, 0.859]	0.731	0.444	[0.704, 0.758]
	Filler	0.215	0.411	[0.19, 0.24]	0.213	0.41	[0.188, 0.238]
Bisyllabic	Target	0.797	0.403	[0.772, 0.822]	0.743	0.437	[0.716, 0.77]
	Filler	0.17	0.376	[0.147, 0.193]	0.172	0.377	[0.149, 0.195]

**Table 4.** Means, standard deviations, and 95% confidence intervals for d’ and beta for bisyllabic and monosyllabic target items for participants exposed to sentences produced with IDS or ADS in Experiment 2

Measure	Syllable	IDS			ADS		
		Mean	SD	CI	Mean	SD	CI
*d*’	Monosyllabic	1.796	0.664	[1.629, 1.963]	1.451	0.588	[1.303, 1.599]
	Bisyllabic	1.849	0.748	[1.661, 2.037]	1.664	0.67	[1.495, 1.833]
beta	Monosyllabic	0.977	0.743	[0.79, 1.164]	1.465	1.222	[1.157, 1.773]
	Bisyllabic	1.435	1.164	[1.142, 1.728]	1.722	1.372	[1.376, 2.068]

**Table 5.** Means, standard deviations, and 95% confidence intervals for word acceptance rates for monosyllabic and bisyllabic target items, presented frequently and infrequently, as well as filler items for participants exposed to sentences produced with IDS or ADS in Experiment 3

Word length	Frequency status	IDS			ADS		
		Mean	SD	CI	Mean	SD	CI
Monosyllabic	Frequent target	0.619	0.488	[0.53, 0.708]	0.46	0.5	[0.371, 0.549]
	Infrequent target	0.558	0.499	[0.465, 0.651]	0.481	0.502	[0.394, 0.568]
	Filler	0.547	0.498	[0.51, 0.584]	0.491	0.5	[0.455, 0.527]
Bisyllabic	Frequent target	0.628	0.485	[0.538, 0.718]	0.597	0.492	[0.511, 0.683]
	Infrequent target	0.628	0.485	[0.538, 0.718]	0.597	0.492	[0.511, 0.683]
	Filler	0.543	0.498	[0.506, 0.58]	0.469	0.499	[0.434, 0.504]

**Table 6.** Means, standard deviations, and 95% confidence intervals for d’ and beta for bisyllabic and monosyllabic items presented frequently and infrequently to participants exposed to sentences produced with IDS or ADS in Experiment 3

Measure	Syllable	Frequency	IDS			ADS		
			Mean	SD	CI	Mean	SD	CI
*d*’	Monosyllabic	Frequent target	0.171	0.561	[−0.084, 0.426]	−0.073	0.662	[−0.359, 0.213]
		Infrequent target	0.044	0.747	[−0.296, 0.384]	0.002	0.391	[−0.167, 0.171]
	Bisyllabic	Frequent target	0.221	0.509	[−0.011, 0.453]	0.349	0.489	[0.138, 0.56]
		Infrequent target	−0.191	0.562	[−0.447, 0.065]	−0.126	0.526	[−0.354, 0.102]
beta	Monosyllabic	Frequent target	0.891	0.213	[0.794, 0.988]	1.034	0.54	[0.801, 1.267]
		Infrequent target	0.887	0.243	[0.776, 0.998]	1.056	0.461	[0.857, 1.255]
	Bisyllabic	Frequent target	0.966	0.238	[0.858, 1.074]	1.005	0.522	[0.779, 1.231]
		Infrequent target	0.966	0.269	[0.844, 1.088]	0.936	0.235	[0.834, 1.038]

## Data Availability

The stimuli, experiment files, data, analysis scripts, and RMarkdown file used in this study to create this article are available on the Open Science Framework (osf.io/f2mqe).
