# Supplementary material for: Ab initio word recognition in infant- and adult-directed continuous speech
Source: Appl Psycholinguist. Author manuscript; Available in PMC 2026 Feb 18. (PMC12818925; doi:10.1017/s0142716425100350)
Supplement: 1 [file NIHMS2123729-supplement-1.pdf]

Appendix

Appendix A

Appendix A contains lists of target and filler items (A1) and sentence lists (A2).

Table A1. List of all target and filler items used in Experiments 1-3, including their word length. All target and filler items appeared in the test phase during Experiments 1 and 3, but not Experiment 2. The target and filler items used in Experiment 2 are indicated.

Word Status	Word Length	Item	Used in Exp. 2
Target	Monosyllabic	Braut	yes
Target	Monosyllabic	Fleiss	yes
Target	Monosyllabic	Gips	
Target	Monosyllabic	Groll	
Target	Monosyllabic	Hengst	
Target	Monosyllabic	Huhn	yes
Target	Monosyllabic	Korb	yes
Target	Monosyllabic	Nuss	yes
Target	Monosyllabic	Priem	yes
Target	Monosyllabic	Rutsch	yes
Target	Monosyllabic	Schub	yes
Target	Monosyllabic	Teig	yes
Target	Bisyllabic	Dorfschmied	
Target	Bisyllabic	Eltern	
Target	Bisyllabic	Hemmnis	
Target	Bisyllabic	Hinblick	yes
Target	Bisyllabic	Ingwer	yes
Target	Bisyllabic	Irrtum	yes
Target	Bisyllabic	Julis	yes
Target	Bisyllabic	Kautschuk	yes
Target	Bisyllabic	Leugnung	yes
Target	Bisyllabic	Missgriff	yes
Target	Bisyllabic	Rindvieh	yes
Target	Bisyllabic	Taeubin	yes
Filler	Monosyllabic	Biss	yes
Filler	Monosyllabic	Bock	yes
Filler	Monosyllabic	Brunn	yes
Filler	Monosyllabic	Durst	yes
Filler	Monosyllabic	Fels	yes
Filler	Monosyllabic	Firn	yes
Filler	Monosyllabic	Flugs	yes

(continued)

Word Status	Word Length	Item	Used in Exp. 2
Filler	Monosyllabic	Glied	yes
Filler	Monosyllabic	Greif	yes
Filler	Monosyllabic	Gruss	yes
Filler	Monosyllabic	Heus	yes
Filler	Monosyllabic	Hirt	yes
Filler	Monosyllabic	Huld	yes
Filler	Monosyllabic	Keil	yes
Filler	Monosyllabic	Kerls	yes
Filler	Monosyllabic	Kluft	yes
Filler	Monosyllabic	Krupp	yes
Filler	Monosyllabic	Laub	yes
Filler	Monosyllabic	Luchs	yes
Filler	Monosyllabic	Mauls	yes
Filler	Monosyllabic	Mulm	yes
Filler	Monosyllabic	Mut	yes
Filler	Monosyllabic	Neid	yes
Filler	Monosyllabic	Prunk	yes
Filler	Monosyllabic	Rausch	yes
Filler	Monosyllabic	Saums	yes
Filler	Monosyllabic	Schreck	yes
Filler	Monosyllabic	Schrott	yes
Filler	Monosyllabic	Senf	yes
Filler	Monosyllabic	Spiess	yes
Filler	Monosyllabic	Suff	yes
Filler	Monosyllabic	Sund	yes
Filler	Monosyllabic	Tisch	yes
Filler	Monosyllabic	Traum	yes
Filler	Monosyllabic	Turm	yes
Filler	Monosyllabic	Wurf	yes
Filler	Bisyllabic	Bildern	yes
Filler	Bisyllabic	Birnbaum	yes
Filler	Bisyllabic	Borretsch	yes
Filler	Bisyllabic	Dolmetsch	yes
Filler	Bisyllabic	Drosslung	yes
Filler	Bisyllabic	Enkel	yes
Filler	Bisyllabic	Erbschein	yes
Filler	Bisyllabic	Faedlein	yes
Filler	Bisyllabic	Faultier	yes
Filler	Bisyllabic	Fenster	yes
Filler	Bisyllabic	Forsche	yes
Filler	Bisyllabic	Fuhrwerk	yes
Filler	Bisyllabic	Futter	yes
Filler	Bisyllabic	Geiseln	yes
Filler	Bisyllabic	Griessbrei	yes

(continued)

Word Status	Word Length	Item	Used in Exp. 2
Filler	Bisyllabic	Haftlings	yes
Filler	Bisyllabic	Hauptschuld	yes
Filler	Bisyllabic	Heidin	yes
Filler	Bisyllabic	Hirnhaut	yes
Filler	Bisyllabic	Kerbschnitt	yes
Filler	Bisyllabic	Kielraum	yes
Filler	Bisyllabic	Mopse	yes
Filler	Bisyllabic	Neigung	yes
Filler	Bisyllabic	Nietung	yes
Filler	Bisyllabic	Tiefsinn	yes
Filler	Bisyllabic	Tierkreis	yes
Filler	Bisyllabic	Torfmull	yes
Filler	Bisyllabic	Trittbrett	yes
Filler	Bisyllabic	Ufers	yes
Filler	Bisyllabic	Ulme	yes
Filler	Bisyllabic	Urlaub	yes
Filler	Bisyllabic	Urteil	yes
Filler	Bisyllabic	Verskunst	yes
Filler	Bisyllabic	Wettlauf	yes
Filler	Bisyllabic	Windel	yes
Filler	Bisyllabic	Wirtin	yes

Table A2. List of all the sentences used in the exposure phases of Experiments 1 and 3, including the target item, its word length (in number of syllables), and in which training set (A, B) the target item was presented frequently (8x). The first two sentences listed in each target item group were the sentences used when the target item was presented infrequently (2x).

Frequent Presentation	Word Length	Item	Sentence
A	2	Dorfschmied	Er ist der Dorfschmied in Heimsheim
A	2		Die Einwohner schaetzen ihren Dorfschmied sehr
A	2		Er lernt damit er Dorfschmied sein kann
A	2		Dieser Dorfschmied hat einen exzellenten Ruf
A	2		Leider kann nicht jeder Dorfschmied sein
A	2		Die Arbeit von diesem Dorfschmied ist sehr anstrengend
A	2		Ein Dorfschmied braucht wenige Hilfsmittel
A	2		Sie hat alles was ein Dorfschmied braucht
A	2	Hemmnis	Das Hemmnis existiert nur in ihrem Kopf
A	2		Diese Aufgabe stellt ein Hemmnis dar
A	2		Was koennte wohl sein Hemmnis sein
A	2		Ihr Hemmnis ist dagegen nicht so schlimm
A	2		Ohne Hemmnis kaemen die frei
A	2		Sie sagte dass dies ihr groesstes Hemmnis sei
A	2		Ohne Hemmnis waeren wir schon laengst fertig
A	2		Das sollte eigentlich kein Hemmnis darstellen
A	2	Hinblick	Im Hinblick darauf haben wir es nicht getan
A	2		Saetze mit Hinblick drin sind kompliziert
A	2		In diesem Hinblick koennen wir uns auf ihm verlassen
A	2		Das Auto ist in dem Hinblick zu gross
A	2		Genauer Hinblick kann Geld sparen
A	2		Bei genauem Hinblick werden wir verlieren
A	2		Sie mag in diesem Hinblick nicht mitkommen
A	2		In dem Hinblick sollten wir das nicht tun
A	2		Sie meint dass es mein Irrtum war
A	2		Es muss sich hierbei um den Irrtum handeln
A	2		Dieser Irrtum wird noch Folgen haben
A	2		Bei dem Irrtum sollte man nicht fortfahren
A	2		Mein Irrtum ist dass ich ihm geglaubt habe

(continued)

Frequent Presentation	Word Length	Item	Sentence
A	2	Irrtum	Sie versicherte mir dass dies ein Irrtum sei
A	2		Sie sagte der Irrtum sei mein Fehler
A	2		Ihr Irrtum war es ihm zu glauben
A	2	Kautschuk	Reifen werden aus Kautschuk hergestellt
A	2		Der meiste Kautschuk kommt aus Suedamerika
A	2		Dieser Kautschuk ist besonders fuer den Sommer geeignet
A	2		Aus Kautschuk kann man nicht nur Gummi herstellen
A	2		Der Kautschuk hat hervorragende Qualitaet
A	2		Zum Schluss wird Kautschuk aufgebracht
A	2		Wir verwenden Kautschuk und Plastik
A	2		Natuerlicher Kautschuk ist billig
A	2	Rindvieh	Ich mag es nicht wenn er mich ein Rindvieh nennt
A	2		Der Farmer hat nur dieses Rindvieh gehabt
A	2		Das Rindvieh hat immer sehr schnell gefressen
A	2		Das liebste Fressen von dem Rindvieh war Heu
A	2		Mein Rindvieh ist braun und weiss gefaerbt
A	2		Ordentliches Rindvieh hat immer ein schoenes Fell
A	2		Die Farbe von ihrem Rindvieh war jedoch hell
A	2		Deshalb war sein Rindvieh wohl krank
A	1	Braut	Die Braut sah in ihrem Kleid absolut bezaubernd aus
A	1		Der Braeutigam der Braut war sehr stolz
A	1		Die rote Braut wird hoffentlich bald da sein
A	1		Am Wegesrand sah man ihre Braut stehen
A	1		Als der Bus ankam sass die Braut auf der Rueckbank
A	1		Der Strauss der Braut lag auf dem Gehweg
A	1		Zum Glueck kam die andere Braut in die Kirche
A	1		Man kann sich bei der Zeitschrift eine Braut bestellen
A	1		Sie hat ihren Fleiss gezeigt
A	1		Ohne Fleiss ist der beste Versuch nichts wert
A	1		Sie wies Fleiss auf bei ihrer Arbeit
A	1		Mein Fleiss hat mich zum Erfolg gefuehrt
A	1		Manche Menschen sagen Fleiss waere die beste Tugend

(continued)

Frequent Presentation	Word Length	Item	Sentence
A	1	Fleiss	Dein Fleiss wird sich am Ende auszahlen
A	1		Man kann Fleiss nicht ersetzen
A	1		Gute Absicht bringt nur mit Fleiss etwas
A	1	Gips	Der Gips ist noch nicht trocken
A	1		Ihre Statue aus Gips stand in der Ecke
A	1		Auch die Wand ist aus Gips gemacht
A	1		Der Gips befindet sich in der Treppe
A	1		Sie wollte ein Vogelhaus mit Gips basteln
A	1		Mit Gips ist es schneller
A	1		Beton und Gips sind alte Stoffe
A	1		Die Maenner haben einigen Gips verwendet
A	1	Groll	Sie hegte grossen Groll gegen ihren Bruder
A	1		Er blickt voller Groll auf die Welt
A	1		Der staendige Groll bescherte ihm ein Magengeschwuer
A	1		Zu wenig Groll ist schlecht
A	1		Menschen hegen Groll aus seltsamen Gruenden
A	1		Empfindest du immer noch Groll wegen dieser Sache
A	1		Damit entfachte er den Groll der ganzen Nation
A	1		Ohne Groll waere er im Leben nicht angekommen
A	1	Hengst	Der Hengst konnte fuer den ganzen Tag laufen
A	1		Auf der Weide standen sein Hengst und ihre Stute
A	1		Heute hat er den schwarzen Hengst erworben
A	1		Der linke Fuss von diesem Hengst ist verletzt
A	1		Sie malte ein Bild von ihrem Hengst im Wald
A	1		Der braune Hengst war sehr stur
A	1		Ein zahmer Hengst ist selten besonders
A	1		Hans ist der einzige Hengst im Stall
A	1		Sie weiss warum das Huhn den Weg kreuzte
A	1		Kam das schwarze Huhn oder das Ei zuerst
A	1		Dieses Huhn legt jeden Tag ein Ei
A	1		Mein Huhn ist sehr laut
A	1		Manches Huhn kann sogar fliegen

(continued)

Frequent Presentation	Word Length	Item	Sentence
A	1	Huhn	Ich habe gehoert dass ein Huhn Maeuse essen kann
A	1		Das Huhn von ihr mochte den Sonnenaufgang
A	1		Isst du lieber Huhn oder Schwein
B	2	Eltern	Meine Eltern kommen mich jeden Monat besuchen
B	2		Ihre Eltern wohnen in Hamburg
B	2		Er hat sehr lange bei seinen Eltern gewohnt
B	2		Die Eltern von ihm sind sehr reich
B	2		Manche Eltern passen nicht auf Ihre Kinder auf
B	2		Er weiss dass ihn die Eltern lieben
B	2		Diese Woche gehen wir meine Eltern besuchen
B	2		Das haben ihre Eltern auch gesagt
B	2	Ingwer	Sie trinkt Tee mit Ingwer um nicht krank zu werden
B	2		Die Pflanze Ingwer waechst sehr schnell
B	2		Er fand den Ingwer zu scharf
B	2		Die Suppe ist mit Ingwer gewuerzt
B	2		Er mag Ingwer nur in moderaten Mengen
B	2		Sie liebt Bonbons ohne Ingwer oder Honig
B	2		Diesen Ingwer habe ich selbst gepflanzt
B	2		Ich kenne manchen dem Ingwer nicht schmeckt
B	2	Julis	Der schoenste Tag des Julis ist der dreiundzwanzigste
B	2		Am Ende dieses Julis wird der August anfangen
B	2		Am Morgen eines Julis fahre ich in die Ferien
B	2		Ich habe schon mehrere schoene Julis erlebt
B	2		Die meisten Julis verbrachte er am See
B	2		Einige Julis waren sehr verregnet
B	2		Die Julis wurden meistens Zuhause verbracht
B	2		Neben dem Fruehling sind Julis die schoenste Jahreszeit
B	2		Ihre Leugnung machte den Prozess sehr schwierig
B	2		Bei den Beschuldigungen hilft alle Leugnung nichts
B	2		Eine Leugnung ist die beste Strategie
B	2		An ihrer Leugnung aendert sich nichts
B	2		Aller Leugnung zum Trotz war er schuldig

(continued)

Frequent Presentation	Word Length	Item	Sentence
B	2	Leugnung	Sie sollte mit Leugnung anfangen
B	2		Danach koennte er Leugnung ausprobieren
B	2		Zum Schluss konnte Leugnung auch nicht helfen
B	2	Missgriff	Ein Missgriff bedeutet den sicheren Tod
B	2		Dieser Erwerb ist ein Missgriff gewesen
B	2		Der Missgriff koennte seiner gewesen sein
B	2		Sein Missgriff hatte schwere Folgen
B	2		Dies ist ihr Missgriff gewesen
B	2		Er warnte dass dies ein grosser Missgriff sei
B	2		Ein grosser Missgriff kann schwere Folgen haben
B	2		Ihr Missgriff war nicht so schlimm
B	2	Taeubin	Ihre liebste Taeubin ist grau
B	2		Die Taeubin sitzt schon lange auf dem Dach
B	2		Manche Taeubin findet keinen Partner
B	2		Nicht jeder kann so schoen wie deine Taeubin aussehen
B	2		Ich sehe es wenn diese Taeubin fliegt
B	2		Die Taeubin flog auf dem Balkon
B	2		Wir haben den ganzen Tag meine Taeubin gesucht
B	2		Seine blaue Taeubin sieht super aus
B	1	Korb	Zum Geburtstag bekam ich einen Korb mit Fruechten
B	1		Mein grosser Korb besteht aus Weidenruten
B	1		Sie gab ihm ihren Korb weil die ihn nicht mochte
B	1		Der Korb ist mit Blumen gefuellt
B	1		Sie nutzt den Korb fuer ihre Arbeit
B	1		Ich habe den Korb neben ihrer Bank vergessen
B	1		Leider schwimmt ihr Korb im Wasser
B	1		Der schwarze Korb wurde heute verloren
B	1		Diese Nuss kann man essen
B	1		Die einzelne Nuss lag neben dem Ast
B	1		Zum Oeffnen der Nuss benoetigt man Hilfsmittel
B	1		Ihre Nuss war schwer zu knacken
B	1		Die fuenfzehner Nuss passt auf diese Schraube

(continued)

Frequent Presentation	Word Length	Item	Sentence
B	1	Nuss	Die Torte war mit Nuss verziert
B	1		Er hat mich eine bloede Nuss genannt
B	1		Sie ass noch ihre letzte Nuss an diesem Abend
B	1	Priem	Sie schwamm an dem Priem vorbei
B	1		Der Priem war voller Wasser
B	1		Sie wollte immer schon den Priem sehen
B	1		Das Schiff war im Priem auf Grund gelaufen
B	1		Ein Priem besteht meistens aus Sand
B	1		Dieser Priem ist mit Pflanzen bewachsen
B	1		Wir wollten den Priem mit ihrem Boot umfahren
B	1		Ich habe noch nicht ihren Priem gesehen
B	1	Rutsch	Wir wuenschen dir einen guten Rutsch ins neue Jahr
B	1		In einem Rutsch erledigt sich die Arbeit am besten
B	1		Mein Rutsch nach Zwanzigsiebzehn war nicht gut
B	1		Wir haben den Rutsch in Berlin gefeiert
B	1		Ich rutsch fuer mein Leben gern
B	1		Dieser Rutsch wird der beste werden
B	1		Ihr Rutsch war dieses Jahr langweilig
B	1		Kein Rutsch war so toll wie dieser
B	1	Schub	Die Maschine gab vollen Schub beim Start
B	1		Der Schub blieb lange Zeit konstant
B	1		Ich gebe Schub um schneller zu fliegen
B	1		Wenig Schub hilft sehr wenig
B	1		Sein Schub ist geringer als ihrer
B	1		Fuenftausend Newton Schub sind stark
B	1		Der Pilot hat den Schub reduziert
B	1		Mit wenig Schub fliegt er zur Insel
B	1		Der Teig fuer die Plaetzchen ist fertig
B	1		Rohen Teig sollte man nicht essen
B	1		Fuer den Kuchen hat er den Teig zubereitet
B	1		Ihre Kinder haben beide Teig stibitzt
B	1		Im Supermarkt kann man fertigen Teig finden

(continued)

Frequent Presentation	Word Length	Item	Sentence
B	1	Teig	Dieser Teig ist allerdings nicht so gut
B	1		Authentischer Teig ist dagegen besser
B	1		Ihr Mann mag es wenn Schokolade in den Teig gepackt wird

Appendix B

Appendix B contains a summary of the transitional probabilities of target items in both training sets.

Table B1. For each target word presented in training set A, information is given on its frequency of presentation, syllable length, average backwards TP at word onset (Word Onset BTP), internal TP, and average forwards TP at word offset (Word Offset FTP). The mean of the transitional probability values is given, with the range in parenthesis.

Frequency	Syllables	Item	Word Onset BTP	Internal TP	Word Offset FTP
Frequent	Monosyllabic	Groll	0.125 (0.125 - 0.125)		0.125 (0.125 - 0.125)
		Hengst	0.125 (0.125 - 0.125)		0.167 (0.125 - 0.25)
		Huhn	0.143 (0.125 - 0.25)		0.143 (0.125 - 0.25)
		Priem	0.143 (0.125 - 0.25)		0.125 (0.125 - 0.125)
		Rutsch	0.125 (0.125 - 0.125)		0.143 (0.125 - 0.25)
		Teig	0.143 (0.125 - 0.25)		0.143 (0.125 - 0.25)
	Bisyllabic	Eltern	0.2 (0.125 - 0.25)	1	0.125 (0.125 - 0.125)
		Hinblick	0.167 (0.125 - 0.25)	1	0.125 (0.125 - 0.125)
		Ingwer	0.143 (0.125 - 0.25)	1	0.125 (0.125 - 0.125)
		Irrtum	0.143 (0.125 - 0.25)	1	0.167 (0.125 - 0.25)
		Kautschuk	0.143 (0.125 - 0.25)	1	0.143 (0.125 - 0.25)
		Taeubin	0.167 (0.125 - 0.25)	1	0.125 (0.125 - 0.125)
Infrequent	Monosyllabic	Braut	0.5 (0.5 - 0.5)		0.5 (0.5 - 0.5)
		Fleiss	0.5 (0.5 - 0.5)		0.5 (0.5 - 0.5)
		Gips	0.5 (0.5 - 0.5)		0.5 (0.5 - 0.5)
		Korb	0.5 (0.5 - 0.5)		0.5 (0.5 - 0.5)
		Nuss	0.5 (0.5 - 0.5)		0.5 (0.5 - 0.5)
		Schub	0.5 (0.5 - 0.5)		0.5 (0.5 - 0.5)
	Bisyllabic	Dorfschmied	0.5 (0.5 - 0.5)	1	0.5 (0.5 - 0.5)
		Hemmnis	0.5 (0.5 - 0.5)	1	0.5 (0.5 - 0.5)
		Julis	0.5 (0.5 - 0.5)	1	0.5 (0.5 - 0.5)
		Leugnung	0.5 (0.5 - 0.5)	1	0.5 (0.5 - 0.5)
		Missgriff	0.5 (0.5 - 0.5)	1	0.5 (0.5 - 0.5)
		Rindvieh	0.5 (0.5 - 0.5)	1	0.5 (0.5 - 0.5)

Table B2. For each target word presented in training set B, information is given on its frequency of presentation, syllable length, average backwards TP at word onset (Word Onset BTP), internal TP, and average forwards TP at word offset (Word Offset FTP). The mean of the transitional probability values is given, with the range in parenthesis.

Frequency	Syllables	Item	Word Onset BTP	Internal TP	Word Offset FTP
Infrequent	Monosyllabic	Groll	0.5 (0.5 - 0.5)		0.5 (0.5 - 0.5)
		Hengst	0.5 (0.5 - 0.5)		0.5 (0.5 - 0.5)
		Huhn	0.5 (0.5 - 0.5)		0.5 (0.5 - 0.5)
		Priem	0.5 (0.5 - 0.5)		0.5 (0.5 - 0.5)
		Rutsch	0.5 (0.5 - 0.5)		0.5 (0.5 - 0.5)
		Teig	0.5 (0.5 - 0.5)		0.5 (0.5 - 0.5)
	Bisyllabic	Eltern	0.5 (0.5 - 0.5)	1	0.5 (0.5 - 0.5)
		Hinblick	0.5 (0.5 - 0.5)	1	0.5 (0.5 - 0.5)
		Ingwer	0.5 (0.5 - 0.5)	1	0.5 (0.5 - 0.5)
		Irrtum	0.5 (0.5 - 0.5)	1	0.5 (0.5 - 0.5)
		Kautschuk	0.5 (0.5 - 0.5)	1	0.5 (0.5 - 0.5)
		Taeubin	0.5 (0.5 - 0.5)	1	0.5 (0.5 - 0.5)
Frequent	Monosyllabic	Braut	0.2 (0.125 - 0.25)		0.125 (0.125 - 0.125)
		Fleiss	0.125 (0.125 - 0.125)		0.125 (0.125 - 0.125)
		Gips	0.2 (0.125 - 0.25)		0.143 (0.125 - 0.25)
		Korb	0.143 (0.125 - 0.25)		0.125 (0.125 - 0.125)
		Nuss	0.125 (0.125 - 0.125)		0.125 (0.125 - 0.125)
		Schub	0.143 (0.125 - 0.25)		0.125 (0.125 - 0.125)
	Bisyllabic	Dorfschmied	0.143 (0.125 - 0.25)	1	0.167 (0.125 - 0.25)
		Hemmnis	0.143 (0.125 - 0.25)	1	0.143 (0.125 - 0.25)
		Julis	0.125 (0.125 - 0.125)	1	0.125 (0.125 - 0.125)
		Leugnung	0.125 (0.125 - 0.125)	1	0.125 (0.125 - 0.125)
		Missgriff	0.2 (0.125 - 0.25)	1	0.143 (0.125 - 0.25)
		Rindvieh	0.125 (0.125 - 0.125)	1	0.167 (0.125 - 0.25)

Appendix C

Appendix C contains the full model results reported in Experiment 1.

Table C1. The results of the general mixed-effects model without an intercept which compared of target item word acceptance to chance. The maximum random effects structure had random intercepts for subjects and items, with a random slope for Word Length on the subjects intercept and Register on the items intercept. Formula: Word_Acceptance ~ -1 + Frequency Status : Register : Word Length + (1 + Word Length | Subject) + (1 + Register | Item).

Parameter	Coefficient	*CIlow*	*CIhigh*	z-value	p-value
Frequency (Freq.) × Word Length (Bisyll.) × Register (IDS)	0.634	0.083	1.185	2.255	0.024
Frequency (Infreq.) × Word Length (Bisyll.) × Register (IDS)	-0.247	-0.793	0.298	-0.888	0.374
Frequency (Freq.) × Word Length (Monosyll.) × Register (IDS)	0.192	-0.449	0.834	0.587	0.557
Frequency (Infreq.) × Word Length (Monosyll.) × Register (IDS)	0.091	-0.552	0.735	0.278	0.781
Frequency (Freq.) × Word Length (Bisyll.) × Register (ADS)	0.211	-0.274	0.697	0.853	0.394
Frequency (Infreq.) × Word Length (Bisyll.) × Register (ADS)	-0.245	-0.739	0.249	-0.972	0.331
Frequency (Freq.) × Word Length (Monosyll.) × Register (ADS)	-0.063	-0.657	0.531	-0.209	0.835
Frequency (Infreq.) × Word Length (Monosyll.) × Register (ADS)	-0.388	-0.986	0.209	-1.274	0.203

Table C2. The results of the general mixed-effects model which examined how the fixed effects Frequency Status (Frequent Target, Infrequent Target, Filler), Register (IDS, ADS) and Word Length (Mono-, Bisyllabic) influenced word acceptance. The random effects structure included random intercepts for subjects and items, with random slopes for Register and Word Length on the subjects intercept. Formula: Word_Acceptance ~ 1 + Register x Word Length x Frequency Status + (1 + Register + Word Length | Subject) + (1 | Item).

Parameter	Coefficient	*CIlow*	*CIhigh*	z-value	p-value
Intercept	0.034	-0.216	0.285	0.270	0.7872
Register	0.143	-0.064	0.351	1.356	0.1752
Frequency Status (Freq.)	0.217	0.052	0.382	2.572	0.0101
Frequency Status (Infreq.)	-0.219	-0.384	-0.053	-2.589	0.0096
Word Length	0.019	-0.163	0.202	0.205	0.8376
Register × Frequency Status (Freq.)	0.036	-0.110	0.181	0.481	0.6307
Register × Frequency Status (Infreq.)	-0.015	-0.161	0.131	-0.204	0.8386
Register × Word Length	-0.048	-0.165	0.069	-0.808	0.4189
Frequency Status (Freq.) × Word Length	0.163	-0.002	0.328	1.936	0.0528
Frequency Status (Infreq.) × Word Length	-0.076	-0.242	0.090	-0.900	0.3681
Register × Frequency Status (Freq.) × Word Length	0.089	-0.057	0.234	1.198	0.2309
Register × Frequency Status (Infreq.) × Word Length	-0.067	-0.213	0.079	-0.901	0.3676

Table C3. The results of the linear mixed-effects model with the intercept removed to examine in which conditions *d'* was above chance. The maximum random effects structure had a random intercept for subjects, with a random slope for Word Length. Formula: D_Prime ~ 0 + Register : Word Length : Frequency + (1 + Word Length | Subject).

Parameter	Coefficient	*CIlow*	*CIhigh*	t-value	p-value
Frequency (Freq.) × Register (IDS) × Word Length (Bisyll.)	0.268	0.052	0.484	2.452	0.015
Frequency (Infreq.) × Register (IDS) × Word Length (Bisyll.)	-0.161	-0.377	0.055	-1.472	0.143
Frequency (Freq.) × Register (ADS) × Word Length (Bisyll.)	0.155	-0.056	0.366	1.451	0.149
Frequency (Infreq.) × Register (ADS) × Word Length (Bisyll.)	-0.092	-0.303	0.118	-0.866	0.388
Frequency (Freq.) × Register (IDS) × Word Length (Monosyll.)	-0.076	-0.320	0.169	-0.613	0.541
Frequency (Infreq.) × Register (IDS) × Word Length (Monosyll.)	-0.083	-0.328	0.161	-0.675	0.501
Frequency (Freq.) × Register (ADS) × Word Length (Monosyll.)	0.002	-0.237	0.241	0.016	0.988
Frequency (Infreq.) × Register (ADS) × Word Length (Monosyll.)	-0.161	-0.400	0.078	-1.331	0.185

Table C4. The results of the linear mixed-effects model which examined how the fixed effects Frequency (Frequent, Infrequent), Register (IDS, ADS) and Word Length (Mono-, Bisyllabic) influenced *d'*. The random effects structure included a random intercept for subjects, with a random slope for Word Length. Formula: Word_Acceptance ~ 1 + Register x Word Length x Frequency + (1 + Word Length | Subject).

Parameter	Coefficient	*CIlow*	*CIhigh*	t-value	p-value
Intercept	-0.019	-0.110	0.073	-0.403	0.6875
Frequency Status (Freq.)	0.106	0.036	0.176	2.996	0.0032
Register	0.006	-0.086	0.097	0.120	0.9043
Word Length	0.061	-0.028	0.150	1.352	0.1784
Frequency Status (Freq.) × Register	0.003	-0.066	0.073	0.093	0.9260
Frequency Status (Freq.) × Word Length	0.063	-0.007	0.133	1.790	0.0753
Register × Word Length	0.006	-0.084	0.095	0.124	0.9013
Frequency Status (Freq.) × Register × Word Length	0.042	-0.028	0.112	1.191	0.2353

Appendix D

Appendix D contains the full model results reported in Experiment 2.

Table D1. The results of the general mixed-effects model without an intercept which compared of target item word acceptance to chance. The maximum random effects structure had random intercepts for subjects and items, with random slopes for Register on both the subjects and items intercepts. Formula: Word_Acceptance ~ -1 + Register : Word Length + (1 + Register | Subject) + (1 + Register | Item).

Parameter	Coefficient	*CIlow*	*CIhigh*	z-value	p-value
Word Length (Bisyll.) × Register (IDS)	1.607	1.148	2.066	6.865	<0.001
Word Length (Monosyll.) × Register (IDS)	1.893	1.429	2.357	7.999	<0.001
Word Length (Bisyll.) × Register (ADS)	1.247	0.705	1.789	4.511	<0.001
Word Length (Monosyll.) × Register (ADS)	1.186	0.645	1.728	4.293	<0.001

Table D2. The results of the general mixed-effects model which examined how the fixed effects Word Status (Target, Filler), Register (IDS, ADS) and Word Length (Mono-, Bisyllabic) influenced word acceptance. The random effects structure included random intercepts for subjects and items, with random slopes for Word Status, Register, and Word Length on the subjects intercept and Word Status and Register in interaction on the items intercept. Formula: Word_Acceptance ~ 1 + Register x Word Length x Word Status + (1 + Register + Word Length + Word Status | Subject) + (1 + Register x Word Status | Item).

Parameter	Coefficient	*CIlow*	*CIhigh*	z-value	p-value
Intercept	-0.073	-0.278	0.133	-0.693	0.4883
Word Status	1.545	1.304	1.786	12.576	<0.001
Register	0.124	0.038	0.210	2.824	0.0047
Word Length	-0.094	-0.261	0.074	-1.097	0.2724
Word Status × Register	0.113	0.042	0.184	3.134	0.0017
Word Status × Word Length	0.044	-0.166	0.254	0.410	0.6817
Register × Word Length	-0.045	-0.126	0.037	-1.070	0.2848
Word Status × Register × Word Length	-0.039	-0.107	0.029	-1.125	0.2604

Table D3. The results of the linear mixed-effects model with the intercept removed to examine in which conditions *d'* was above chance. The maximum random effects structure had a random intercept for subjects, with a random slope for Word Length. Formula: D_Prime ~ 0 + Register : Word Length + (1 | Subject).

Parameter	Coefficient	*CIlow*	*CIhigh*	t-value	p-value
Register (IDS) × Word Length (Bisyll.)	1.849	1.685	2.014	22.087	<0.001
Register (ADS) × Word Length (Bisyll.)	1.664	1.499	1.829	19.877	<0.001
Register (IDS) × Word Length (Monosyll.)	1.796	1.631	1.961	21.452	<0.001
Register (ADS) × Word Length (Monosyll.)	1.451	1.286	1.616	17.333	<0.001

Table D4. The results of the linear mixed-effects model which examined how the fixed effects Register (IDS, ADS) and Word Length (Mono-, Bisyllabic) influenced *d'*. The random effects structure included a random intercept for subjects. Formula: Word_Acceptance ~ 1 + Register x Word Length + (1 | Subject).

Parameter	Coefficient	$CIlow$	$CIhigh$	t-value	p-value
(Intercept)	1.690	1.559	1.821	25.435	<0.001
Register	0.133	0.075	0.190	4.506	<0.001
Word Length	0.067	0.009	0.124	2.263	0.025
Register × Word Length	-0.040	-0.098	0.018	-1.358	0.176

Appendix E

Appendix E contains the full model results reported in Experiment 3.

Table E1. The results of the general mixed-effects model without an intercept which compared of target item word acceptance to chance. The maximum random effects structure had random intercepts for subjects and items. Formula: Word_Acceptance ~ -1 + Frequency Status : Register : Word Length + (1 | Subject) + (1 | Item).

Parameter	Coefficient	*CIlow*	*CIhigh*	z-value	p-value
Frequency (Freq.) × Word Length (Bisyll.) × Register (IDS)	0.541	0.062	1.019	2.214	0.027
Frequency (Infreq.) × Word Length (Bisyll.) × Register (IDS)	-0.122	-0.583	0.339	-0.519	0.604
Frequency (Freq.) × Word Length (Monosyll.) × Register (IDS)	0.485	0.018	0.953	2.036	0.042
Frequency (Infreq.) × Word Length (Monosyll.) × Register (IDS)	0.264	-0.204	0.732	1.105	0.269
Frequency (Freq.) × Word Length (Bisyll.) × Register (ADS)	0.441	-0.012	0.893	1.909	0.056
Frequency (Infreq.) × Word Length (Bisyll.) × Register (ADS)	-0.442	-0.901	0.017	-1.887	0.059
Frequency (Freq.) × Word Length (Monosyll.) × Register (ADS)	-0.156	-0.608	0.296	-0.678	0.498
Frequency (Infreq.) × Word Length (Monosyll.) × Register (ADS)	-0.099	-0.544	0.346	-0.435	0.663

Table E2. The results of the general mixed-effects model which examined how the fixed effects Frequency Status (Frequent Target, Infrequent Target, Filler), Register (IDS, ADS) and Word Length (Mono-, Bisyllabic) influenced word acceptance. The random effects structure included random intercepts for subjects and items, with a random slope for Word Length on the subjects intercept. Formula: Word_Acceptance ~ 1 + Register x Word Length x Frequency Status + (1 + Word Length | Subject) + (1 | Item).

Parameter	Coefficient	*CIlow*	*CIhigh*	z-value	p-value
Intercept	0.088	-0.150	0.325	0.724	0.469
Register	0.184	-0.009	0.378	1.866	0.062
Frequency Status (Freq.)	0.250	0.085	0.415	2.968	0.003
Frequency Status (Infreq.)	-0.206	-0.369	-0.042	-2.469	0.014
Word Length	-0.018	-0.200	0.165	-0.189	0.850
Register × Frequency Status (Freq.)	0.018	-0.130	0.167	0.244	0.807
Register × Frequency Status (Infreq.)	0.009	-0.138	0.156	0.124	0.901
Register × Word Length	-0.040	-0.161	0.082	-0.639	0.523
Frequency Status (Freq.) × Word Length	0.205	0.040	0.370	2.439	0.015
Frequency Status (Infreq.) × Word Length	-0.190	-0.354	-0.027	-2.280	0.023
Register × Frequency Status (Freq.) × Word Length	-0.094	-0.243	0.054	-1.243	0.214
Register × Frequency Status (Infreq.) × Word Length	0.029	-0.118	0.176	0.384	0.701

Table E3. The results of the linear mixed-effects model with the intercept removed to examine in which conditions *d'* was above chance. The maximum random effects structure had a random intercept for subjects, with a random slope for Word Length and Frequency. Formula: D_Prime ~ 0 + Register : Word Length : Frequency + (1 + Word Length + Frequency | Subject).

Parameter	Coefficient	*CIlow*	*CIhigh*	t-value	p-value
Frequency (Freq.) × Register (IDS) × Word Length (Bisyll.)	0.221	0.008	0.433	2.049	0.042
Frequency (Infreq.) × Register (IDS) × Word Length (Bisyll.)	-0.191	-0.436	0.054	-1.543	0.125
Frequency (Freq.) × Register (ADS) × Word Length (Bisyll.)	0.349	0.146	0.552	3.391	<0.001
Frequency (Infreq.) × Register (ADS) × Word Length (Bisyll.)	-0.126	-0.360	0.107	-1.067	0.288
Frequency (Freq.) × Register (IDS) × Word Length (Monosyll.)	0.171	-0.071	0.413	1.399	0.164
Frequency (Infreq.) × Register (IDS) × Word Length (Monosyll.)	0.044	-0.204	0.292	0.350	0.727
Frequency (Freq.) × Register (ADS) × Word Length (Monosyll.)	-0.073	-0.304	0.158	-0.625	0.533
Frequency (Infreq.) × Register (ADS) × Word Length (Monosyll.)	0.002	-0.234	0.239	0.020	0.984

Table E4. The results of the linear mixed-effects model which examined how the fixed effects Frequency (Frequent, Infrequent), Register (IDS, ADS) and Word Length (Mono-, Bisyllabic) influenced *d'*. The random effects structure included a random intercept for subjects, with random slopes for Word Length and Frequency. Formula: Word_Acceptance ~ 1 + Register x Word Length x Frequency + (1 + Word Length + Frequency | Subject).

Parameter	Coefficient	*CIlow*	*CIhigh*	t-value	p-value
Intercept	0.050	-0.038	0.137	1.117	0.2656
Frequency Status (Freq.)	0.117	0.031	0.204	2.675	0.0082
Register	0.012	-0.076	0.099	0.261	0.7941
Word Length	0.014	-0.072	0.099	0.311	0.7561
Frequency Status (Freq.) × Register	0.017	-0.069	0.104	0.397	0.6922
Frequency Status (Freq.) × Word Length	0.104	0.038	0.170	3.125	0.0021
Register × Word Length	-0.060	-0.146	0.026	-1.380	0.1695
Frequency Status (Freq.) × Register × Word Length	-0.033	-0.099	0.033	-0.996	0.3206
